# Lipid metabolism analysis reveals that DGAT1 regulates Th17 survival by controlling lipid peroxidation in uveitis

**DOI:** 10.1172/jci.insight.184072

**Published:** 2025-04-08

**Authors:** Tianfu Wang, Runping Duan, Zhaohuai Li, Bowen Zhang, Qi Jiang, Loujing Jiang, Jianjie Lv, Wenru Su, Lei Feng

**Affiliations:** 1 State Key Laboratory of Ophthalmology, Zhongshan Ophthalmic Center, Sun Yat-sen University, Guangdong Provincial Key Laboratory of Ophthalmology and Visual Science, Guangdong Provincial Clinical Research Center for Ocular Diseases, Guangzhou, China.; 2 Department of Clinical Medicine, Zhongshan School of Medicine, Sun Yat-Sen University, Guangzhou, China.; 3 Eye center, The Second Affiliated Hospital, Zhejiang University School of Medicine, Hangzhou, China.

**Keywords:** Autoimmunity, Immunology, Ophthalmology, Autoimmune diseases, T cells

## Abstract

Lipid metabolism is closely linked with antitumor immunity and autoimmune disorders. However, the precise role of lipid metabolism in uveitis pathogenesis is not clear. In our study, we analyzed the single-cell RNA-Seq (scRNA-Seq) data from cervical draining lymph nodes (CDLNs) of mice with experimental autoimmune uveitis (EAU), revealing an increased abundance of fatty acids in Th17 cells. Subsequent scRNA-Seq analysis identified the upregulation of DGAT1 expression in EAU and its marked reduction under various immunosuppressive agents. Suppression of DGAT1 prevented the conversion of fatty acids into neutral lipid droplets, resulting in the accumulation of lipid peroxidation and subsequent reduction in the proportion of Th17 cells. Inhibiting lipid peroxidation by Ferrostatin-1 effectively restored Th17 cell numbers that were decreased by DGAT1 inhibitor. Moreover, we validated the upregulation of DGAT1 in CD4^+^ T cells from patients with Vogt-Koyanagi-Harada (VKH) disease, a human uveitis. Inhibiting DGAT1 induced lipid peroxidation in human CD4^+^ T cells and reduced the proportion of Th17 cells. Collectively, our study focused on elucidating the regulatory mechanisms underlying Th17 cell survival and proposed that targeting DGAT1 may hold promise as a therapeutic approach for uveitis.

## Abstract 2

A lipid-lowering agent could suppress the progression of uveitis by regulating intracellular lipid peroxidation levels and modulating Th17 cell survival.

## Graphical abstract

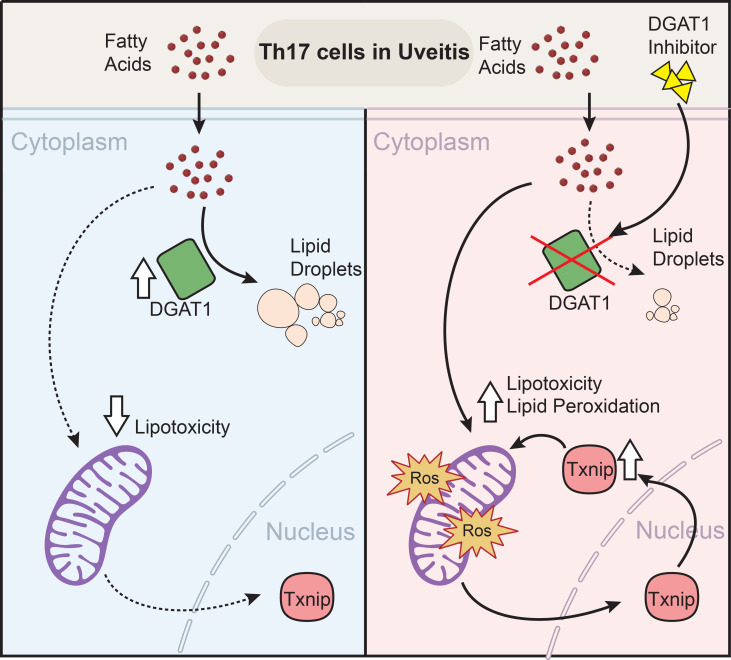

## Introduction

Uveitis, representing a spectrum of immune-mediated intraocular pathologies, accounts for about 10%–15% of blindness in the world (1–3). Although the exact etiology remains elusive, accumulated evidence demonstrates that the dysregulation of Th17 cells plays a crucial role in driving autoimmunity in uveitis (4). Unfortunately, effective treatments are limited.

Lipids consist of fatty acids, phospholipids, and cholesterol. Reprogramming of lipid metabolism has been reported to alter immune cell activation, differentiation, and function (5). Lipids are essential for membrane synthesis during clonal expansion and various cellular processes, such as signaling and energy homeostasis, in effector T cells (6, 7). Exogenous supplementation of long-chain fatty acids (FAs) enhances the differentiation of Th17 cells (8–10). Moreover, lipid metabolism is closely linked with autoimmune disorders, such as systemic lupus erythematosus (11) and inflammatory bowel disease (12). Elevated triglyceride levels have been observed in patients with uveitis (13). However, it is not clear how lipid dysregulation modulates the Th17 cell metabolism and, thus, participates in uveitis pathogenesis. A better understanding of the mechanism governing the differentiation and function of Th17 cells, particularly their survival, is required.

Lymph nodes (LNs) are pivotal in activating T cells and initiating autoimmune responses (14–17). Effector T cells engaged in autoimmunity, originating from LNs, migrate to target organs, inducing inflammation and tissue damage (16, 18). Among these nodes, cervical draining LNs (CDLNs) hold particular significance as major CNS-draining LNs (19). The removal of CDLNs prior to the induction of experimental autoimmune encephalomyelitis — a paradigmatic model closely resembling experimental autoimmune uveitis (EAU), which is a widely accepted animal model of uveitis, in pathogenic mechanisms (20) — leads to a marked reduction in disease severity (21).

In this work, we analyzed the published single-cell RNA-Seq data (scRNA-Seq) data from CDLNs of EAU mice from the perspective of lipid metabolism, revealing a heightened abundance of FAs in Th17 cells. Then we compared the single-cell transcriptomes from EAU in response to multiple immunosuppressive agents and identified the upregulation of DGAT1 expression in EAU and its marked reduction in response to various immunosuppressive agents. Inhibiting DGAT1 alleviated EAU, with notably reducing the Th17 cell proportion and increasing the Treg proportion. Suppression of DGAT1 prevented the conversion of FAs into neutral lipid droplets (LDs), resulting in the accumulation of lipid peroxidation and consequent reduction in the proportion of Th17 cells. Inhibiting lipid peroxidation by Ferrostatin-1 (Fer-1) effectively restored Th17 cell numbers, which were decreased by the DGAT1 inhibitor. Furthermore, we verified the upregulation of DGAT1 in CD4^+^ T cells in a human uveitis, Vogt-Koyanagi-Harada (VKH) disease. Inhibiting DGAT1 also induced lipid peroxidation in human CD4^+^ T cells and diminished the proportion of Th17 cells in patients with VKH. With the investigation into the lipid metabolism of Th17 cells, our work focused on the regulatory mechanism underlying Th17 cell survival and suggested that targeting DGAT1 may be a promising therapy for uveitis.

## Results

### Th17 cells in EAU mice exhibited a higher abundance of FAs.

**Figure 1:**
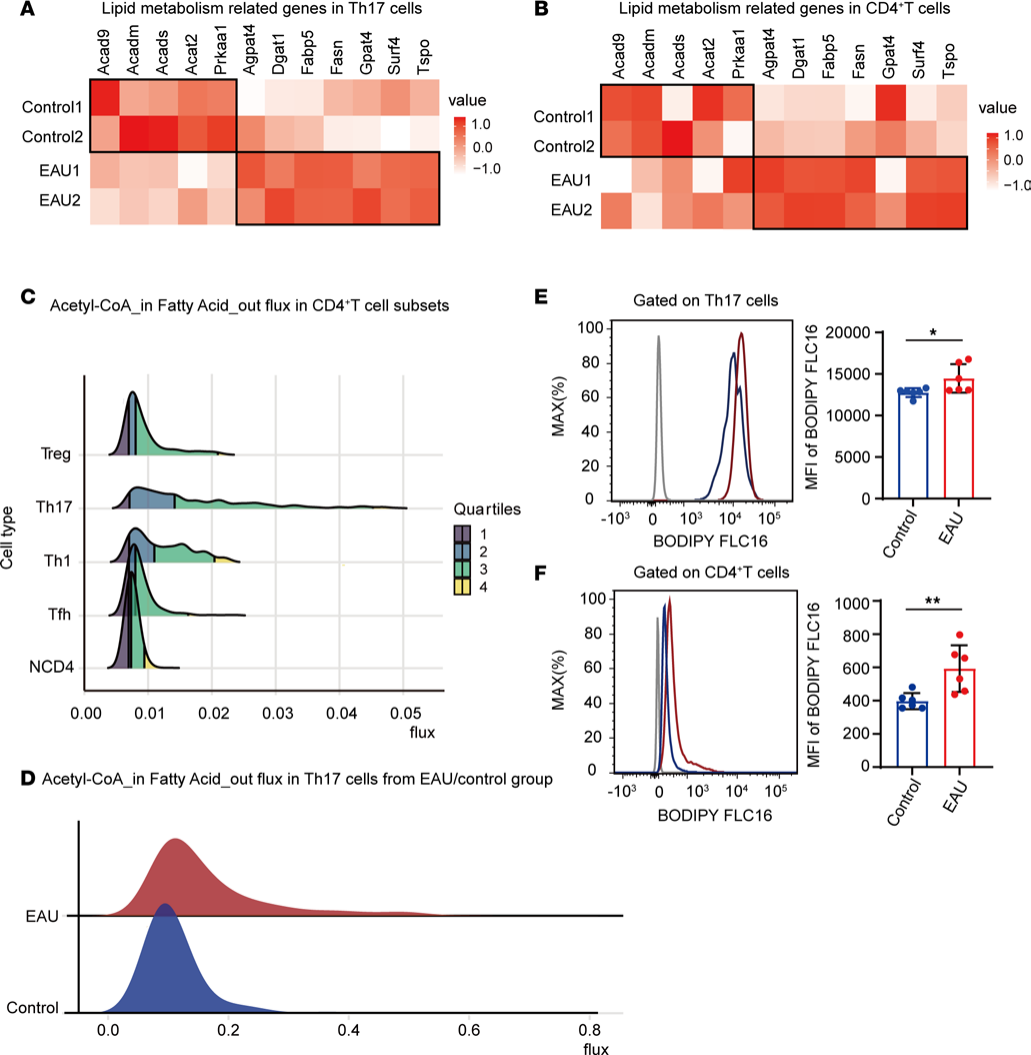
Th17 cells in EAU mice exhibited a higher abundance of fatty acids. (**A** and **B**) Heatmap showing lipid metabolism related genes of Th17 cells (**A**) and CD4^+^ cells (**B**) in the EAU/control comparison groups. (**C**) Ridgeline plots indicating the distribution values of metabolic flux in Treg, Th17, Th1, TFH, and NCD4 cells. Each ridgeline represents the flux of Acetyl-CoA to Fatty Acid, shown on the *x* axis, for different CD4^+^ T cell subsets, shown on the *y* axis. The computational method scFEA was used to infer cell-wise fluxome from the scRNA-Seq data to predict the metabolic profiling as well as the differential metabolite conversion rate in cells that correspond to a metabolic flux value. (**D**) Ridgeline plot indicates the distribution values of metabolic flux in Th17 cells from the EAU and the control groups. Each ridgeline represents the flux of Acetyl-CoA to fatty acid and shown on the *x* axis. (**E** and **F**) Representative histograms and mean fluorescence intensity (MFI) values of BODIPY FLC16 in Th17 cells (**E**) and CD4^+^ T cells (**F**) (*n* = 6, data are presented as mean ± SD, significance was determined using unpaired 2-tailed Student’s *t* test). *P < 0.05, **P < 0.01. EAU, experimental autoimmune uveitis.

Hallmarks of T cell activation involve increased uptake of metabolic substrates and a shift from catabolic to anabolic metabolism (22). For deeper insights into the lipid metabolism transcriptomes in uveitis, we analyzed the published scRNA-Seq data of our lab from the CDLNs of EAU mice. In Th17 cells from the EAU group, genes associated with lipid catabolism (23), such as *Acad9*, *Acadm*, *Acads*, *Acat2*, and *Prkaa1*, exhibited a declining trend, while key genes linked to the synthesis of FAs (*Fasn*, *Gpat4*), FAs uptake (*Fabp5*), synthesis of triglycerides (*Agpat4*, *Dgat1*), and transport of cholesterol (*Surf4*, *Tspo*) (24) showed an upregulation trend (Figure 1A). CD4^+^ T cells displayed a comparable pattern except specific genes (Acads, *Acat2*, *Prkaa1* and *Gpat4*) within the aforementioned gene set (Figure 1B). In contrast, the expression pattern of these genes in Th1 cells exhibited less pronounced changes compared with those in Th17 cells (Supplemental Figure 1; supplemental material available online with this article; https://doi.org/10.1172/jci.insight.184072DS1).

Th17 cells heavily rely on FA biosynthesis (9). Inhibiting FA biosynthesis significantly suppresses Th17 cell differentiation (10). To explore the metabolic flux in CD4^+^ T cell subsets from EAU, we employed single-cell fluxomic estimation analysis (scFEA) (25) and revealed that Th17 cells exhibited higher metabolic activity in the pathway from Acetyl CoA to FAs compared with the other CD4^+^ T cell subsets (Figure 1C). Then, we evaluated the same metabolic pathway in Th17 cells from EAU and control groups, unveiling a higher flux in the EAU group (Figure 1D). These scFEA findings confirmed the enhanced FA synthesis in CD4^+^ T cells and Th17 cells from EAU. Targeting FA metabolism, we further examined the FA uptake and noted a significant increase in external FA uptake by Th17 cells from EAU mice measured by flow cytometry (BODIPY FLC16) (Figure 1E and Supplemental Figure 16A). Similarly, CD4^+^ T cells from EAU mice also displayed elevated external FA uptake (Figure 1F). Results suggest a notable promotion of FA synthesis and FA uptake in Th17 cells from EAU.

### DGAT1 was rescued following the administration of immunosuppressive drugs.

**Figure 2:**
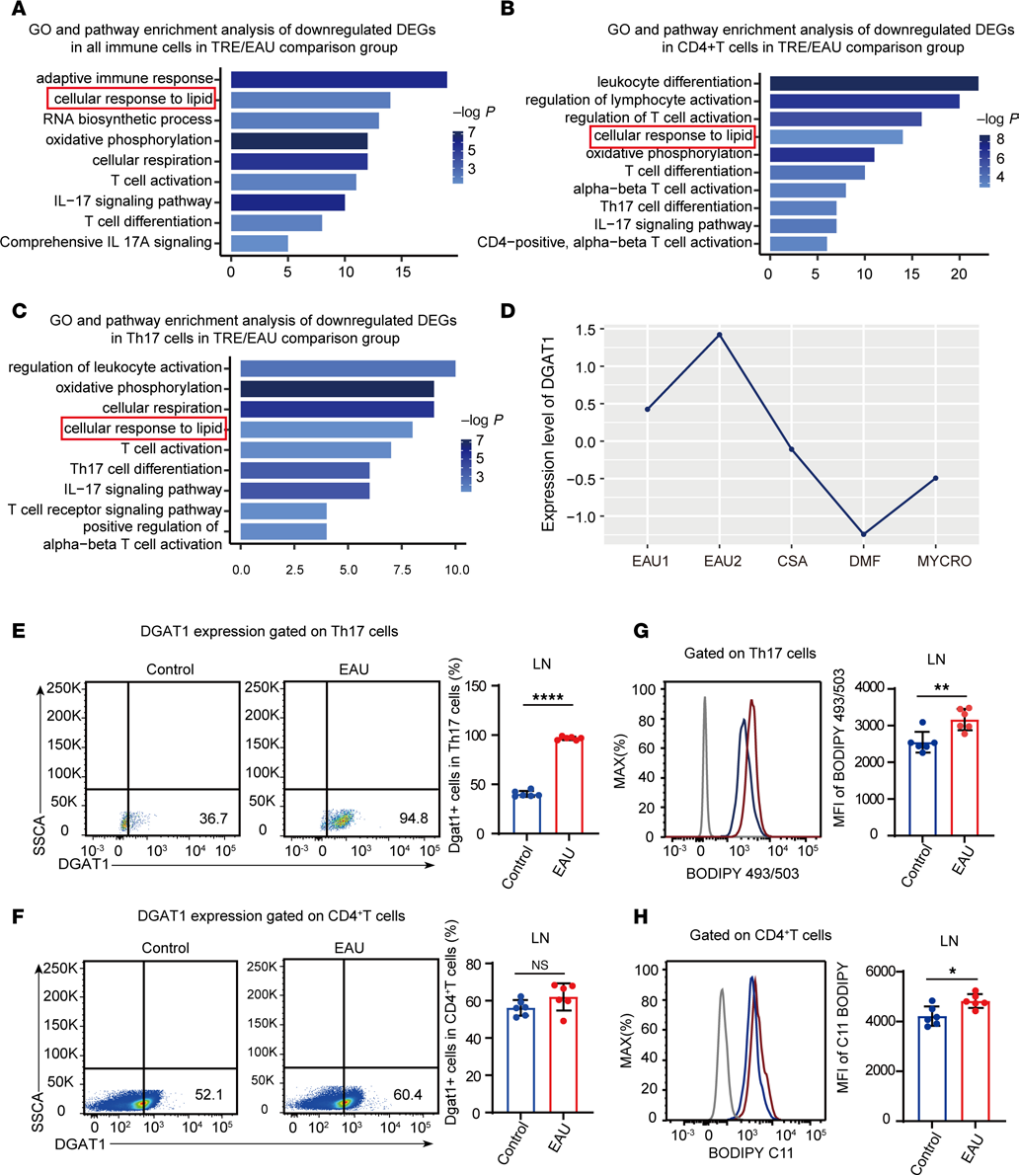
DGAT1 was rescued following the administration of immunosuppressive drugs. (**A**–**C**) Representative GO terms and KEGG pathways enriched in downregulated DEGs of total immune cells (**A**), CD4^+^ T cells (**B**), and Th17 cells (**C**) in the TRE/EAU comparison group. (**D**) Heatmap showing expression levels of DEGs related to lipid metabolism sets. (**E** and **F**) Comparison of DGAT1 expression in Th17 cells (**E**) and CD4^+^ T cells (**F**) between control group and EAU group, measured by flow cytometry after immunization (*n* = 6, data are presented as mean ± SD, significance was determined using unpaired 2-tailed Student’s *t* test). (**G** and **H**) Representative histograms and MFI values of BODIPY 493/503 (**G**) and BODIPY 581/591 C11 (**H**) in Th17 cells (*n* = 6, data are presented as mean ± SD, significance was determined using unpaired 2-tailed Student’s *t* test). *P < 0.05, **P < 0.01, ****P < 0.0001. TRE, immunosuppressive treatment group; EAU, experimental autoimmune uveitis; MYCRO, mycophenolate mofetil; DMF, dimethyl fumarate; CSA, cyclosporine.

To further elucidate the central role of lipid metabolism driving autoimmunity in uveitis, we compared the single-cell transcriptomes of EAU under different immunosuppressive agents, including cyclosporine, dimethyl fumarate, and mycophenolate mofetil. We integrated 7 sets of sequencing data and identified 8 major immune cell types, including T cells, B cells, NK cells, classical DCs (cDC), plasmacytoid DCs (pDC), neutrophils (Neu), monocytes (Mono), and macrophages (Macro) by utilizing classical lineage markers (26, 27) (Supplemental Figure 2, A–C). We classified all 3 immunosuppressive treatment groups as the TRE group and performed gene functional analysis based on the differentially expressed genes (DEGs) between the EAU control and TRE groups. The results showed a marked downregulation of pathways related to the cellular response to lipid and an enrichment of pathways associated with programmed cell death, cellular senescence, and apoptosis in the treatment group (Figure 2A and Supplemental Figure 2D).

Considering the pivotal role of T cells in the pathogenesis of uveitis, we further categorized T cells into 9 subsets, including naive CD4^+^ T cells (NCD4), Treg, Th17, proliferative T cells (ProT), Th1, T follicular helper cells (Tfh), γδ T cells (γδT), CD8^+^ T cells with cytotoxic activity (CTL), and naive CD8^+^ T cells (NCD8) (Supplemental Figure 3, A–C). Similarly, both CD4^+^ T cells and Th17 cells in the TRE group exhibited a downregulation in the cellular response to lipid pathway, along with the pathways related to IL-17 signaling, T cell receptor signaling, and T cell activation (Figure 2, B and C). Further individual analysis of each immunosuppressant revealed a consistent downregulation of the specific lipid metabolism pathway (cellular responses to lipids), aligning with the findings from the TRE/EAU comparison group (Supplemental Figure 4, A–I). Next, we compared the average expression levels of the genes related to lipid metabolism in Th17 cells across all samples, identifying the upregulation of DGAT1 expression in EAU and its marked reduction in response to immunosuppressive drugs (Figure 2D and Supplemental Figure 3D).

Excessive FAs accumulating within the cellular cytoplasm can generate harmful bioactive lipids and disrupts cellular function, ultimately leading to its demise (28, 29). The formation of neutral LDs is considered pivotal in preventing FAs from inducing lipid peroxidation and causing adverse effects on the organism (30). DGAT1 is an integral membrane protein that is highly expressed in the epithelial cells of the small intestine (31). It catalyzes the esterification of FAs into triglycerides, making itself a crucial enzyme in the process of LD formation (32). Through flow cytometry analysis, we observed a marked upregulation of DGAT1 expression in Th17 cells during EAU (Figure 2E and Supplemental Figure 16B), while there was no obvious difference in DGAT1 expression gated on the entire CD4^+^ T cells (Figure 2F). To further evaluate the function of DGAT1, we stained Th17 cells with a neutral lipid dye (BODIPY 493/503) to quantify neutral LDs (33). Th17 cells displayed notably increased staining with BODIPY 493/503, indicative of significantly increased LD formation during EAU (Figure 2G). In addition, consistent with the higher abundance of FAs, cellular lipotoxicity assessed using the fluorescent lipid peroxidation sensor (BODIPY 581/591 C11) (34) was slightly elevated in Th17 cells from the EAU group compared with the control group (Figure 2H).

### Inhibiting DGAT1 alleviated EAU.

**Figure 3:**
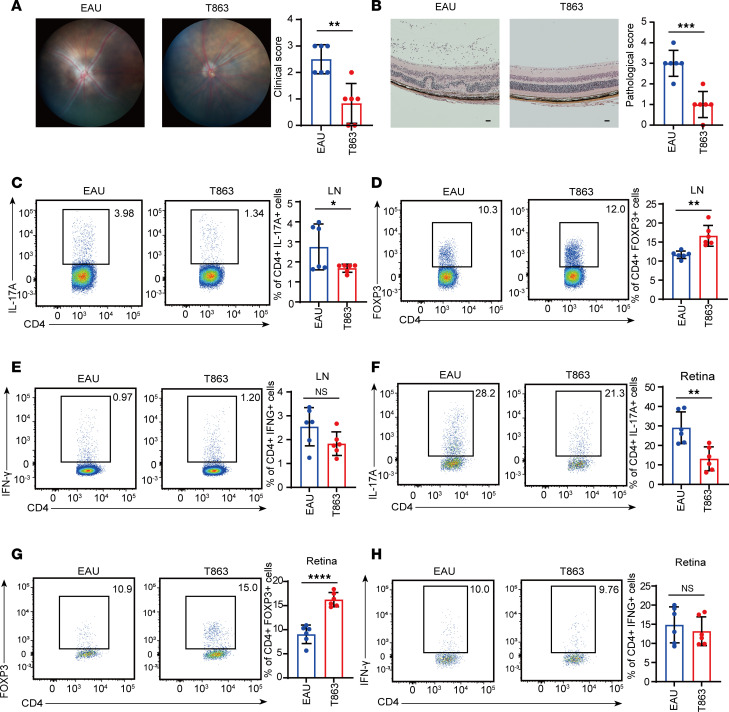
Inhibiting DGAT1 alleviated EAU. (**A**) Representative fundus images and clinical scores of eyes from the EAU group and T863 group (*n* = 6, data are presented as mean ± SD, significance was determined using unpaired 2-tailed Student’s *t* test). (**B**) Representative histopathological images (H&E staining) and pathological scores of eyes from the EAU mice and T863 treated mice(*n* = 6, data are presented as mean ± SD, significance was determined using unpaired 2-tailed Student’s *t* test). Scale bars: 20 μm. (**C**–**E**) Immune cells from the lymph nodes of EAU mice and T863 treated mice. Flow cytometry was performed to show the proportion of Th17 cells (**C**), Tregs (**D**), and Th1 cells (**E**) (*n* = 6, data are presented as mean ± SD, significance was determined using unpaired 2-tailed Student’s *t* test). (**F**–**H**) Immune cells infiltrating the retina from the EAU group and T863 group, Flow cytometry was performed to show the proportion of Th17 cells (**F**), Tregs (**G**), and Th1 cells (**H**) (*n* = 6, data are presented as mean ± SD, significance was determined using unpaired 2-tailed Student’s *t* test). Data are presented as mean ± SD. *P < 0.05, **P < 0.01, ***P < 0.001, ****P < 0.0001. EAU, experimental autoimmune uveitis.

To assess the role of DGAT1 in EAU pathogenesis, we administered T863, a DGAT1 inhibitor, to EAU mice. The inhibitor treatment significantly alleviated EAU symptoms, as evidenced by fundus and histopathological examination (Figure 3, A and B). While the proportion of Th1 cells remained unchanged, T863 notably decreased the proportion of Th17 cell subsets and concurrently increased the population of Tregs within the CDLNs (Figure 3, C–E). Moreover, T863 treatment led to a corresponding decrease in the proportion of Th17 cells within the CD4^+^ T cells infiltrating the retina, accompanied by an increase in the proportion of Tregs, as observed in CDLNs (Figure 3, F–H). These results indicate that inhibiting DGAT1 restored the Th17/Treg balance, emphasizing DGAT1 as a promising therapeutic target for EAU treatment.

### T863 induced functional changes in the immune profile of CDLNs.

**Figure 4:**
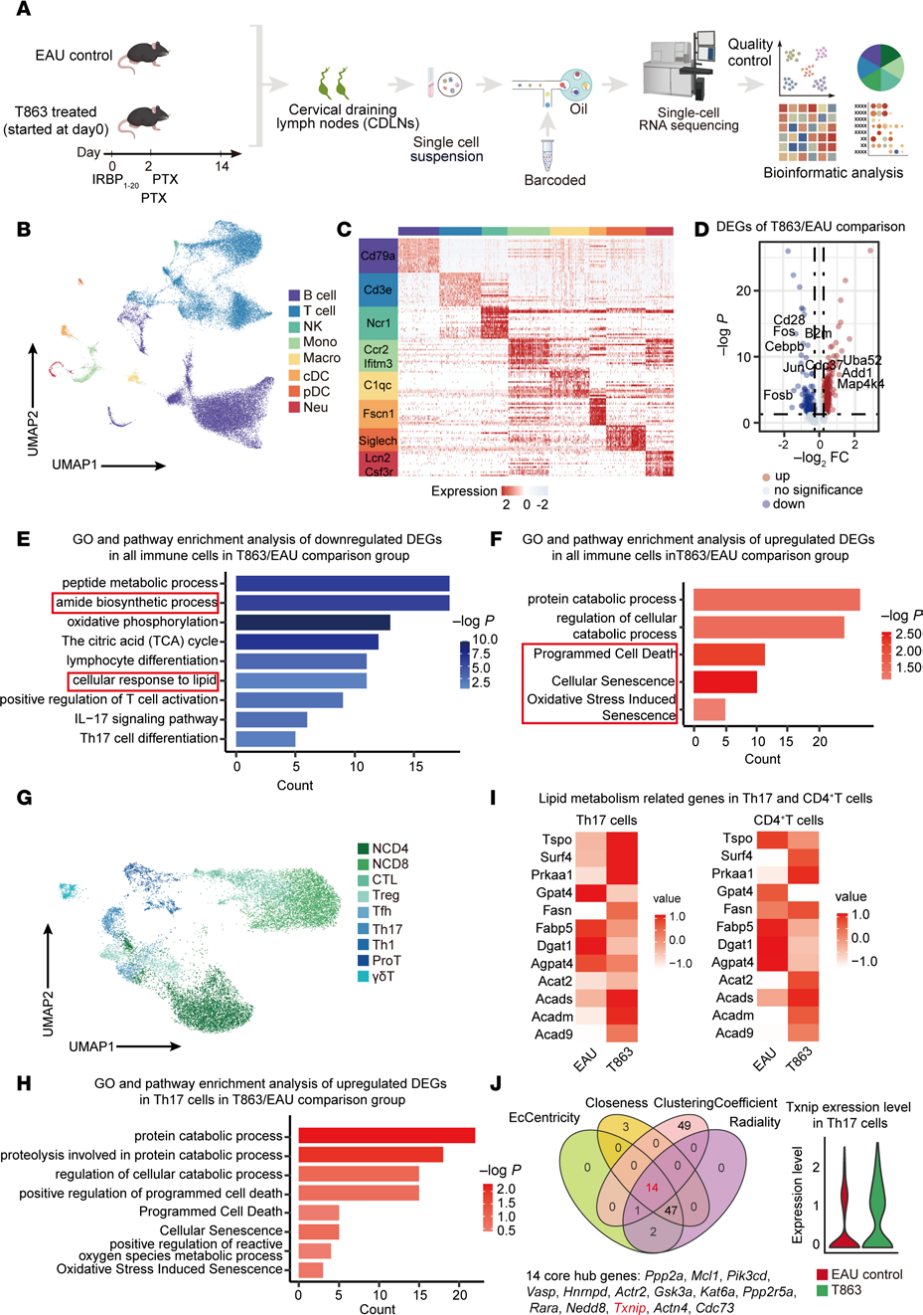
T863 induced functional changes in the immune profile of CDLNs. (**A**) Schematic of the experimental design for scRNA-Seq analysis. CDLN cells were harvested from EAU groups or T863-treated groups at day 14. Each sample included 3 mice. (**B**) UMAP plot showing clusters of CDLN cells from all mice groups. (**C**) Heatmap showing scaled expression of discriminative gene sets for major immune cell types in CDLN cells from all mice groups. (**D**) Volcano plot showing representative DEGs of total immune cells in the T863/EAU comparison groups. Red and blue dots indicate upregulated and downregulated DEGs in T863 groups compared with EAU groups, respectively. Significance was determined using“FindMarkers”function of Seurat package with Wilcoxon rank sum test and adjusted by Bonferroni correction. (**E**) Representative GO terms and KEGG pathways enriched in downregulated DEGs of all immune cells in the T863/EAU comparison group. (**F**) Representative GO terms and KEGG pathways enriched in upregulated DEGs of all immune cells in the T863/EAU comparison group. (**G**) UMAP plot showing clusters of T cells from all mice groups. (**H**) Representative GO terms and KEGG pathways enriched in upregulated DEGs of Th17 cells in the T863/EAU comparison group. (**I**) Heatmap showing lipid metabolism related genes of Th17 cells and CD4^+^ T cells in the T863/EAU comparison groups. (**J**) Venn diagrams showing the number of hub core upregulated DEGs in T863 groups compared with EAU groups (left). Violin plots of *Txnip* in Th17 cell subsets from all mice groups (right).

To further investigate the mechanism underlying the relief from EAU by the DGAT1 inhibitor, we established the T863-treatment EAU model. On day 14, we collected CDLNs from these mice and isolated single-cell suspensions. These suspensions were subsequently utilized to construct barcoded scRNA-Seq libraries for further investigation (Figure 4A).

After clustering immune cell type (Figure 4, B and C, and Supplemental Figure 5A), we performed a DEG analysis to identify the gene signature alterations. The T863 group showed downregulated genes related to IL-17 signaling (*Cebpb*, *Fos*, *Jun*, *Fosb*) and T cell activation (*Cd28*, *B2m*), whereas genes associated with oxidative stress (*Uba52*, *Map4k4*) and cell death (*Add1*, *Cdc37*) were upregulated (Figure 4D). Further gene function analysis showed that pathways involved in Th17 differentiation, IL-17 signaling, cellular response to lipid, amide biosynthetic process, and peptide biosynthetic process were downregulated in T863 group, and pathways associated with programmed cell death, cellular senescence, oxidative stress induced senescence, protein catabolic process, and cellular catabolic process were enriched in T863 group (Figure 4, E and F).

Subsequently, T cells were reclustered into 9 subsets (Figure 4G and Supplemental Figure 6, A and B). To investigate the functional changes in the CD4^+^ T cells, a DEG analysis of all CD4^+^ T cells was conducted. We noticed that genes related to IL-17 signaling (*Fos*, *Fosb*, *Cebpb*, *Id2*, *Jun*) and T cell activation (*B2m*, *Cd2*8) exhibited marked downregulation in the T863 group; genes related to oxidative stress (*Cdk4*, *Mapkapk2*, *Tnik*) showed upregulation, consistent with the findings observed in all immune cell types (Supplemental Figure 6C).

To examine the lipid metabolic changes in CD4^+^ T cells in response to T863 treatment, we conducted gene function analysis. Pathways linked to cellular response to lipid, T cell differentiation, IL-17 signaling, and peptide biosynthetic process were downregulated in CD4^+^ T cells treated with T863 (Supplemental Figure 7A). Subsequent analysis in Th17 cells revealed enrichment in pathways associated with programmed cell death, cellular senescence, positive regulation of ROS metabolic process, and oxidative stress–induced senescence in the T863 group, while pathways related to peptide biosynthetic process, amide biosynthetic process, and DNA biosynthetic process were downregulated (Figure 4H and Supplemental Figure 7B). Then, we examined the expression of genes involved in lipid metabolism. In the T863 group, Th17 cells exhibited a downward trend of genes linked to FAs uptake (*Fabp5*) and synthesis of triglycerides (*Agpat4* and *Dgat1*), while genes related to lipid catabolism (*Acad9*, *Acadm*, *Acads*, *Acat2*, and *Prkaa1*) and transport of cholesterol (*Surf4* and *Tspo*) exhibited an upregulated trend. In CD4^+^ T cells from the T863 group, the expression of these genes showed similar trends (Figure 4I).

Results indicate that the DGAT1 inhibitor induced functional changes in CDLNs, affecting both lipid metabolism and immune-related pathways. In summary, comparing the T863-induced immune profile with the conventional immunosuppressive treatment transcriptomes, we noted the upregulation of pathways related to “oxidative stress” in the T863 group, which were not enriched in the TRE group (Supplemental Figure 7C). This finding was consistent with the known effects of DGAT1. Thus, we reanalyzed the upregulated DEGs in the T863/EAU comparison group and identified 14 core hub genes, namely, *Ppp2a*, *Mcl1*, *Pik3cd*, *Vasp*, *Hnrnpd*, *Actr2*, *Gsk3a*, *Kat6a*, *Ppp2r5a*, *Rara*, *Nedd8*, *Txnip*, *Actn4*, and *Cdc73* from the top 65 hub genes using the cytoHubba plugin with 4 algorithms: EcCentricity, Closeness, ClusteringCoefficient, and Radiality. Of them, *Txnip* was deeply connected with cellular redox regulation (35). Subsequent violin plot intuitively demonstrated its increased expression in Th17 cells in response to T863 (Figure 4J), suggesting that Txnip may be an important factor mediating the effects of DGAT1 inhibition.

### DGAT1 regulated Th17 cell survival via lipid peroxidation.

**Figure 5:**
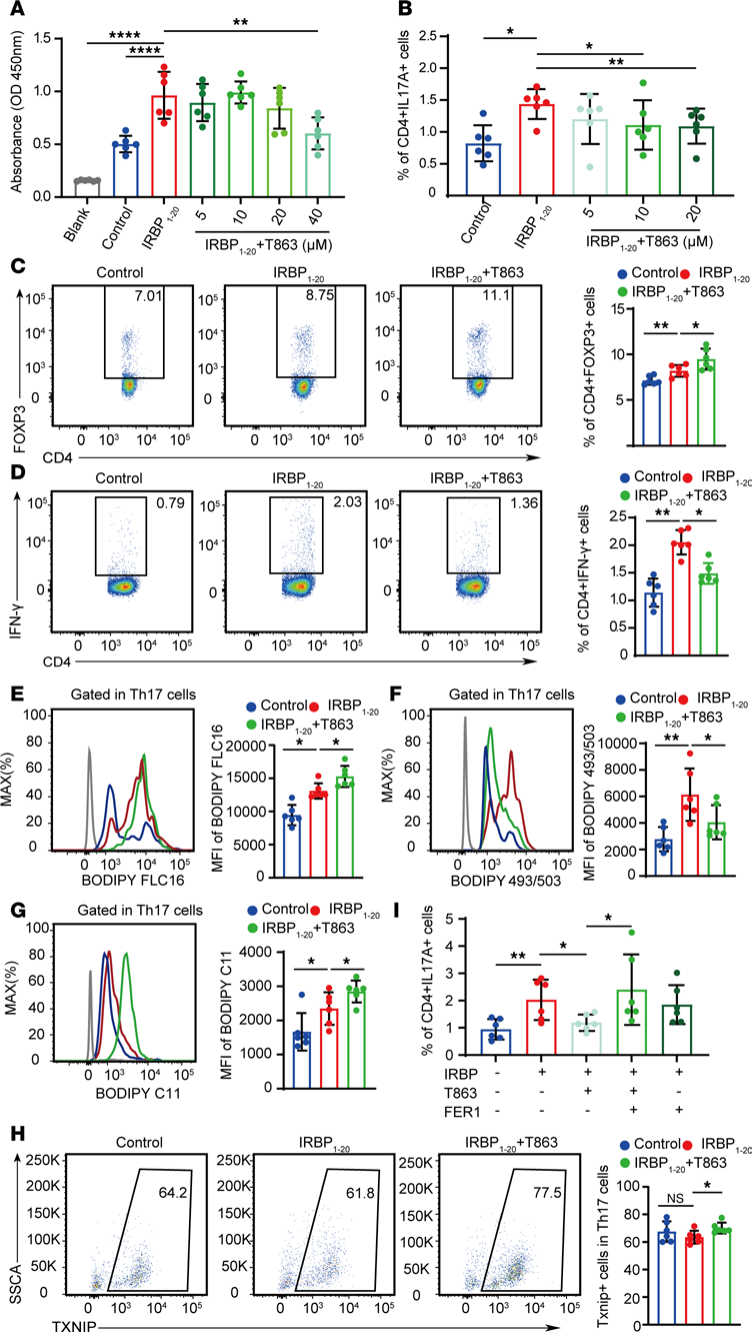
DGAT1 regulated Th17 cell survival via lipid peroxidation. (**A**) CCK8 assay of CDLN cells from EAU mice cultured with no IRBP_1-20_, IRBP_1-20_ alone, or with IRBP_1-20_ with escalating doses of T863 (0–40 μM) for 72 hours, the absorbance of each sample was measured at 450 nm using an automated ELISA reader. (**B**) CD4^+^ T cells of CDLN cells from EAU mice cultured with no IRBP_1-20_, IRBP_1-20_ alone, or with IRBP_1-20_ with escalating doses of T863 (0–20 μM) for 72 hours. Flow cytometry showed the proportion of IL-17A^+^ cells (*n* = 6, data are presented as mean ± SD, significance was determined using 1-way ANOVA). (**C** and **D**) CD4^+^ T cells of CDLN cells from EAU mice cultured with no IRBP_1-20_, IRBP_1-20_ alone, or with IRBP_1-20_ with T863 for 72 hours. Flow cytometry showed the proportion of FOXP3^+^ cells (**C**) and IFN-γ^+^ cells (**D**) in total CD4^+^ gated T cells (*n* = 6, data are presented as mean ± SD, significance was determined using 1-way ANOVA). (**E**–**G**) Representative histograms and MFI values of BODIPY FLC16 (**E**), BODIPY 493/503 (**F**), and BODIPY 581/591 C11 (**G**) in Th17 cells from CDLN cells of EAU mice cultured with T863 for 72 hours (*n* = 6, data are presented as mean ± SD, significance was determined using 1-way ANOVA). (**H**) Th17 cells of CDLN cells from EAU mice cultured with no IRBP_1-20_, IRBP_1-20_ alone, or with IRBP_1-20_ with T863 for 72 hours. Flow cytometry showed the proportion of Txnip^+^ cells in Th17 cells (*n* = 6, data are presented as mean ± SD, significance was determined using 1-way ANOVA). (**I**) CD4^+^ T cells of CDLN cells from EAU mice cultured with/without IRBP, with/without T863, with/without FER1, and cultured for 72 hours. Proportions of CD4^+^IL-17A^+^ cells were measured by flow cytometry (*n* = 6, data are presented as mean ± SD, significance was determined using 1-way ANOVA). Data are presented as mean ± SD. *P < 0.05, **P < 0.01, ****P < 0.0001.

To further investigate the function of DGAT1 in EAU, we conducted in vitro experiment using T863. T863 at 20 μM did not affect cell viability (Figure 5A), and when we cultured CDLNs isolated from EAU mice with interphotoreceptor retinal-binding protein (1–20 amino acid fragment; IRBP_1-20_ for 72 hours in the presence or absence of T863, this dosage effectively suppressed the proportion of Th17 cells (Figure 5B and Supplemental Figure 8A). Therefore, we chose this dose for further experiments, which also reduced the proportion of Th1 cells while increasing that of Tregs (Figure 5, C and D). Lipid peroxidation products 4-HNE and malondialdehyde (MDA) could impede T cell activation (36, 37). As expected, T863 restrained CD4^+^ T cell activation by decreasing expression of activation markers CD25 and CD69 (Supplemental Figure 9, A and B). Meanwhile, we transferred T863-treated CD4^+^ T cells into mice and observed that T863 treatment attenuated the pathogenicity of CD4^+^ T cells (Supplemental Figure 9, C and D). Adenovirus-mediated DGAT1 knockdown revealed comparable alterations in the distribution of CD4^+^ T cell subsets to those observed with T863 treatment (Supplemental Figure 10, A–D), and further adoptive transfer experiments effectively inhibited the induction of EAU by CD4^+^ T cells (Supplemental Figure 11, A and B). Conversely, overexpression of DGAT1 in vitro resulted in opposite trends in CD4^+^ T cell subset proportions (Supplemental Figure 12, A–D) and EAU phenotypes (Supplemental Figure 13, A and B).

Excessive lipid peroxidation is an executive threat to metabolically active cells. For example, heightened lipid peroxidation in T_FH_ cells triggers ferroptosis, leading to a subsequent reduction in T_FH_ cell numbers (38). Maintaining lipid homeostasis is important for tumor growth, since the correct conversion from FAs to LDs protects against excessive lipid peroxidation to avoid lipotoxicity in multiple types of cancer cells (39, 40). Moreover, a recent study has highlighted a biomaterial that was capable of enhancing lipid peroxidation, significantly improving the therapeutic efficiency of radiotherapy in tumors (41). Considering the potential treatment implications, we hypothesize that inhibiting DGAT1 could effectively enhance lipid peroxidation in Th17 cells, making this mechanism a potential therapeutic target in uveitis. In vitro experiments revealed that T863 treatment reduced the frequency of CD4^+^Ki67^+^ cells, indicative of restrained CD4^+^ T cell proliferation (Supplemental Figure 9E). Moreover, T863 application effectively decreased LD formation labeled by BODIPY 493/503, while it markedly increased the levels of FAs and lipid peroxidation in Th17 cells (Figure 5, E–G). However, in Tregs, this trend did not achieve statistical significance (Supplemental Figure 14, A–C).

Thioredoxin interacting protein (Txnip) functions as an important physiological inhibitor of the major antioxidant protein thioredoxin (Trx) (42). Its transcription and activity levels play a major role in cellular redox regulation (43). Excessive ROS triggers the upregulation of Txnip expression and its translocation from the nucleus to the cytoplasm (44). The elevated expression of Txnip antagonizes the antioxidant effects of Trx, thus contributing to further increased ROS level (45). Given the elevated transcript levels of Txnip observed in Th17 cells treated with T863, in Th17 cells, we conducted in vitro experiments and observed a significant elevation of Txnip expression at the protein level following T863 treatment (Figure 5H). The amplification effect of TXNIP on ROS signals might establish a cellular positive feedback loop that accelerates the Th17 cell death. Subsequently, we verified this mechanism in Th17 cells with the commonly used ferroptosis inhibitor, Fer-1, which inhibits cellular ferroptosis by repressing lipid peroxidation (46). Although Fer-1 alone could not alter the proportion of Th17 cells, it effectively rescued the Th17 cell numbers in the presence of T863 (Figure 5I and Supplemental Figure 8B). These results collectively suggest that DGAT1 regulated Th17 cell survival via lipid peroxidation.

### Validating the function of DGAT1 in human VKH disease.

VKH disease is a leading cause of panuveitis in Asia (47, 48). This systemic autoimmune disorder presents as bilateral uveitis often accompanied by neurological, auditory, and integumentary symptoms (49). To explore alterations in the transcriptomic profile and ascertain the preservation of DGAT1 upregulation in human uveitis, we analyzed scRNA-Seq data from peripheral blood mononuclear cells (PBMCs) collected from 6 healthy controls (HC) and patients with VKH. We distinguished 8 distinct immune cell types using established lineage markers (Supplemental Figure 15, A–C). Gene function analysis demonstrated enrichment of pathways annotated as “T cell receptor signaling pathway”, “regulation of CD4-positive, alpha-beta T cell differentiation” and “cellular response to lipid” in VKH datasets (Supplemental Figure 15D).

**Figure 6:**
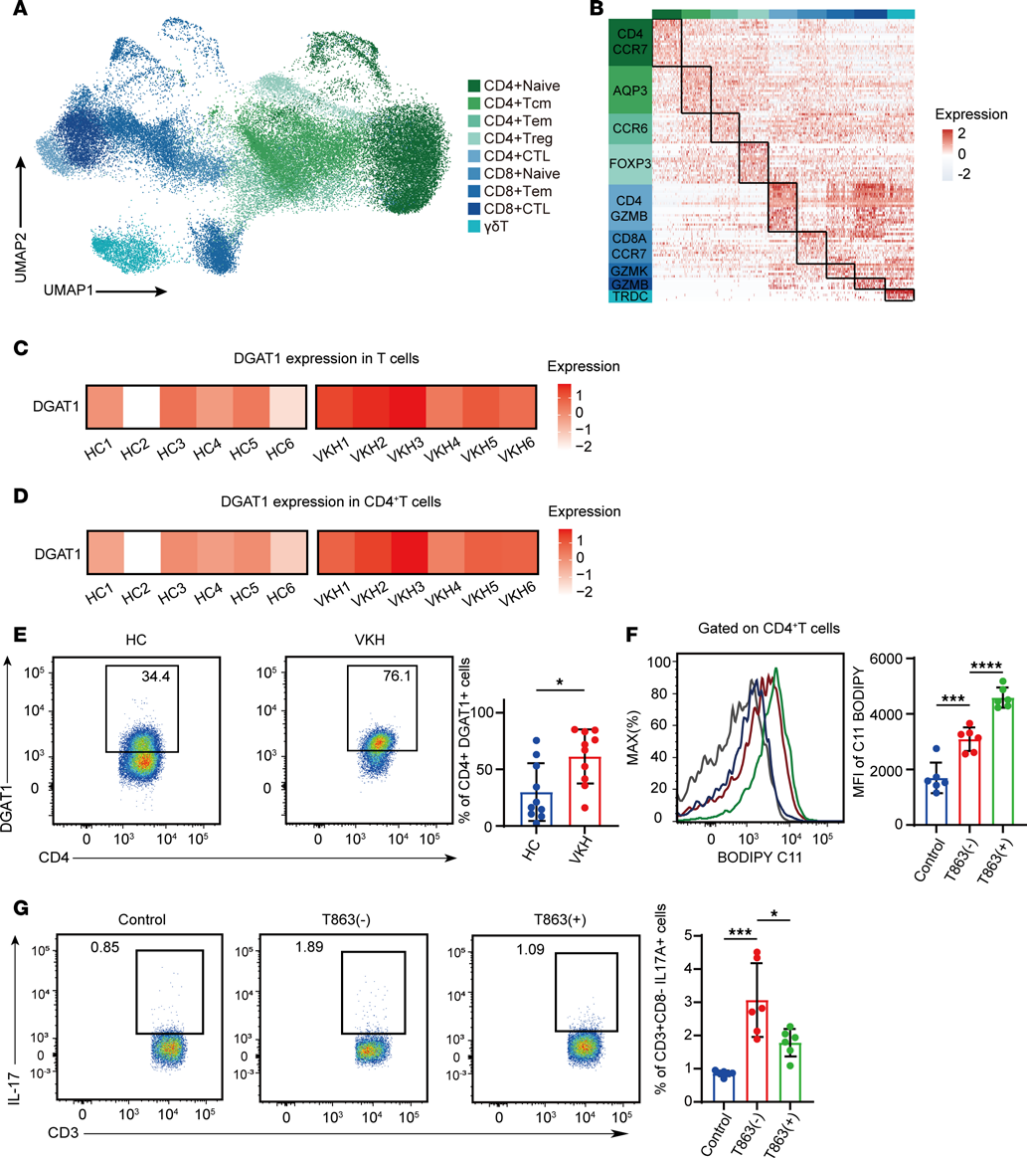
Validating the function of DGAT1 in human VKH disease. (**A**) UMAP plot showing clusters of T cells from VKH group and HC group. (**B**) Heatmap showing scaled expression of discriminative gene sets for major cell types in T cells from VKH group and HC group. (**C** and **D**) Heatmap of the relative expression in Th17 cells (**C**) and CD4^+^ T cells (**D**) in the DGAT1 VKH group and HC group. (**E**) Proportions of CD4^+^DGAT1^+^ cells in patients with VKH and HC were measured by flow cytometry(*n* = 10, data are presented as mean ± SD, significance was determined using unpaired 2-tailed Student’s *t* test). (**F**) Representative histograms and MFI values of BODIPY 581/591 C11 in CD4^+^ cells (*n* = 6, data are presented as mean ± SD, significance was determined using 1-way ANOVA). (**G**) The percentage of Th17 cells in PBMCs treated with or without T863 group, measured by flow cytometry, gated on CD3^+^CD8^–^ cells. Data represented as mean ± SD form. Significance was determined using 1-way ANOVA. *P < 0.05, ***P < 0.001, ****P < 0.0001. Tcm, central memory T cells; Tem, effector memory T cells; CTL, cytotoxic T cells; VKH, Vogt-Koyanagi-Harada disease; HC, healthy controls.

Next, we reclustered T cells and identified 9 known T cell subsets, namely CD4^+^ naive T (CD4^+^ naive) cells, CD4^+^ effector memory T (CD4^+^ Tem) cells, CD4^+^ central memory T (CD4^+^ Tcm) cells, CD4^+^ Tregs, CD8^+^ Tem cells, CD8^+^ cytotoxic T cells (CD8^+^ CTL), CD8^+^ naive T (CD8^+^ naive) cells, and γδT cells (Figure 6, A and B, and Supplemental Figure 15E). *DGAT1* displayed increased expression in the whole T cells and CD4^+^ T cells from patients with VKH (Figure 6, C and D). Pathways involving peptide biosynthetic processes, cellular response to cytokine stimuli, IL-17 signaling pathways, and cellular responses to lipid were enriched in CD4^+^ T cells from the VKH (Supplemental Figure 15F). Flow cytometry analysis further confirmed significantly higher DGAT1 expression levels in CD4^+^ T cells from active patients with VKH compared with HCs (Figure 6E). To validate the function of DGAT1 in human PBMCs, we applied the DGAT1 inhibitor in vitro and found that CD4^+^ T cells treated with T863 showed increased levels of lipid peroxidation (Figure 6F). Correspondingly, T863 effectively repressed the proportion of Th17 cells (Figure 6G). Collectively, these results validated the function of DGAT1 in human CD4^+^ T cells and suggested that therapeutic strategies targeting lipid peroxidation and DGAT1 may offer promise for VKH treatment.

## Discussion

Through our experiments, we integrated several single-cell transcriptomes of EAU under different immunosuppressive agents and analyzed the lipid metabolism characteristics of immune cells during EAU pathogenesis. Th17 cells from EAU exhibited a notable promotion of FA synthesis and FA uptake. Next, we identified the upregulation of DGAT1 expression in EAU and its marked reduction in response to immunosuppressive agents. Subsequent flow cytometry confirmed it as a crucial enzyme in the process of Th17 intracellular LD formation during EAU. Moreover, inhibition of DGAT1 could alleviate EAU. Mechanistically, when DGAT1 was inhibited, the accumulated FAs led to cellular lipotoxicity, causing an increase in intracellular lipid peroxidation and inducing Th17 cell death. Moreover, the elevation in DGAT1 expression and its regulatory role were preserved in human uveitis, specifically VKH. Thus, DGAT1 emerges as a promising therapeutic target for the treatment of uveitis.

Lipids are essential metabolites of cells. FAs are absorbed or synthesized from carbohydrates and are then transformed into neutral lipids, destined for storage in the form of LDs (50). Previous research has emphasized the prevalence of LDs in various cancer types, including glioblastoma (40), hepatocellular carcinoma (51), and renal cell carcinoma (52); there, they play a crucial role in maintaining cellular lipid homeostasis. Immune cells also show a high abundance of LDs. During immune responses triggered by pathogens or autoantigens, immune cells undergo rapid proliferation and differentiation, necessitating increased lipogenic activity to supply essential components and energy (53). Nevertheless, the precise role of LDs in T cell–mediated autoimmunity remains unclear. In this study, using scFEA, we confirmed a metabolic pathway from Acetyl CoA to FAs, indicative of enhanced FA synthesis in Th17 cells. Then, by flow cytometry analysis, we intuitively demonstrated the increased uptake of FAs and elevated LD formation in activated Th17 cells, indicating the potential of lipid metabolism in understanding the pathogenesis of uveitis.

Among the subset of Th cells, Th17 cells are recognized for their pivotal involvement in the development of diverse autoimmune conditions, including but not limited to psoriasis, rheumatoid arthritis, inflammatory bowel disease, steroid-resistant asthma, and multiple sclerosis (54, 55). In order to meet the biosynthetic demands in autoimmune disorders, T cells undergo a critical shift from catabolic to anabolic metabolism (22). Lipid metabolites substantially influence the differentiation and function of Th17 cells under pathological conditions (24). However, compared with well-established knowledge that Th17 cell differentiation and pathogenicity are deeply connected with lipid metabolites, it remains unclear whether a specific pathway controls the survival of Th17 cells. DGAT1 is a polytopic endoplasmic reticulum membrane protein catalyzing the mammalian triacylglycerol synthesis (56, 57), playing a critical role in LD formation by converting FAs into triglycerides (32). The expression pattern and function of DGAT1 have rarely been studied in autoimmune disorders, especially in Th17 cells. In this study, we revealed that DGAT1 expression is increased in Th17 cells from CDLNs of EAU mice. In addition, CD4^+^ T cells from patients with VKH also exhibited heightened DGAT1 expression. Inhibiting DGAT1 alleviated EAU, while notably reducing the Th17 cell proportion. Furthermore, inhibiting DGAT1 blocked the conversion from FAs to LDs and induced excess lipid peroxidation within Th17 cells, resulting in a marked decrease in the proportion of Th17 cells. Our work suggests DGAT1 as a promising therapeutic target for uveitis treatment.

The relationship between lipid metabolism and uveitis has been documented in a previous study (58). This study revealed through a lipidomic analysis that triglycerides can stimulate the proliferation and differentiation of CD4^+^ T cells. Furthermore, a TAG inhibitor (A922500) was proven to impede triglyceride synthesis and alleviate EAU progression. The study suggested that A922500’s effect in inhibiting EAU might be linked to the apoptotic protein Pik3r2. In contrast, by analyzing the scRNA-Seq of EAU from a lipid metabolism perspective, we identified the unique expression pattern of DGAT1 in the Th17 cells. We precisely validated the upregulation of DGAT1 expression in Th17 cells during EAU, and its consistent decrease under multiple immunosuppressants. Then we confirmed it at the protein level, suggesting that DGAT1 expression is associated with the abnormal expansion of Th17 cells. Furthermore, we verified the aberrant FA uptake, LD synthesis, and lipid peroxidation levels at the Th17 cell level, highlighting the core role of DGAT1 in the altered metabolic pattern of Th17 cells. Finally, we demonstrated a marked increase in DGAT1 expression in the PBMCs’ CD4^+^ T cells from patients with VKH, confirming its similar function in humans. Therefore, our study elucidated the function of DGAT1 from the perspective of lipid metabolism regulation; moreover, we found that the therapeutic function of DGAT1 inhibition might extend to human uveitis as well.

ROS acts as an critical signaling molecule in T cells for activation, expansion, and effector function (59, 60), but excessive ROS disrupts redox equilibrium, leading to aberrant cell death (61, 62). Lipid peroxidation involves a complex cascade in which excess ROS target carbon-carbon double bonds in lipids, initiating their oxidative degradation, lipid peroxides formation, and subsequent alterations in the cellular environment (63). It is involved in the regulation of immune cell survival (38). Additionally, recent studies suggest a critical role of lipid peroxidation in several immune-mediated pathological processes, including Leishmania major parasite infection (64) and antitumor immunity (65). Recent research has highlighted a biomaterial capable of enhancing lipid peroxidation, and substantially improved therapeutic efficiency of radiotherapy in tumors (41). Moreover, targeting oxidative stress could selectively eliminate pathogenic cells. Pharmacologic ascorbate exploits differences in oxidative stress, providing radioprotection to normal cells while sensitizing pancreatic cancer cells to radiation (66). Our study demonstrated that inducing lipid peroxidation by inhibiting DGAT1 effectively reduced the proportion of Th17 cells, along with the upregulation of Txnip. The elevated Txnip expression could antagonize the antioxidant effects of Trx, thereby exacerbating the elevation of ROS levels (45). When we treated CDLNs with the commonly used ferroptosis inhibitor, Fer-1, which inhibits cellular ferroptosis by repressing lipid peroxidation (46), the Th17 cell numbers were effectively restored in the presence of DGAT1 inhibitor. These findings together suggested that DGAT1 modulated Th17 cell survival through lipid peroxidation. In addition, targeting lipid peroxidation in Th17 cells presented a potentially novel therapeutic approach for uveitis.

In our study, the inhibition of DGAT1 led to an upregulation of the proportion of Tregs. Our in vitro analysis of lipid metabolism in Tregs revealed that, while drug treatment induced some trends in changes in free FA levels and neutral LD content, these alterations did not reach statistical significance. Furthermore, no significant changes in lipid peroxidation levels were observed. Therefore, DGAT1 inhibition does not appear to affect Treg proportions through the regulation of lipid metabolism. Although a reduction in Th17 cell proportions does not directly result in an increase in Treg proportions, multiple immunoregulatory pathways may influence Treg dynamics. For example, Mbd2 is crucial in promoting Foxp3 expression and Treg suppressive function (67). Studies indicate that targeting Mbd2 reduces Treg numbers and impairs their suppressive function both in vitro and in vivo. Our scRNA-Seq data further demonstrate a significant upregulation of Mbd2 expression in Tregs following T863 treatment (Supplemental Figure 17A). Additionally, Pierson et al. have shown that Mcl-1 is vital for Treg survival, with its absence leading to a rapid loss of Tregs and the onset of severe autoimmunity (68). Following T863 treatment, the transcriptional level of Mcl1 in Tregs from mouse LNs was significantly elevated (Supplemental Figure 17B). The activation of the paracaspase MALT1 is also essential for Treg functionality, as its protease activity regulates TCR-induced upregulation of the transcription factor MYC and the expression of MYC target genes in Tregs. Research has demonstrated that MALT1 protease mediates the connection between TCR signaling and MYC regulation in Tregs, thereby orchestrating metabolism and Treg expansion to maintain immune homeostasis (69). Similarly, we observed a significant increase in Malt1 expression levels in Tregs after T863 administration (Supplemental Figure 17C). These findings provide potential insights into the mechanisms driving the observed increase in Treg proportions.

In summary, by conducting gene functional and lipid metabolism analysis on scRNA-Seq data of EAU mice and immunosuppressive drug–treated EAU mice, we identified DGAT1 as a key modulator of lipid metabolism in Th17 cells. Inhibition of DGAT1 alleviated EAU by preventing the conversion of FAs into neutral LDs, leading to the accumulation of lipid peroxidation and subsequent decrease of Th17 cell survival. Additionally, we confirmed the upregulation of DGAT1 in CD4^+^ T cells from patients with VKH. Inhibiting DGAT1 also induced lipid peroxidation in human CD4^+^ T cells and reduced the proportion of Th17 cells in patients with VKH.

## Methods

### Sex as a biological variable.

Sex was not considered as a biological variable. The study involved both male and female mice, with no significant sex differences observed between the groups. Moreover, the human samples in this study comprised both biological males and females, with no sex-based differences observed between the groups.

### Mice.

The mice used in this study included both male and female mice, and there was no sex difference between the groups. C57BL/6J mice of WT strain, aged 6–8 weeks, were obtained from the Guangzhou Animal Testing Center (Guangzhou, China).

### Human donors.

The human samples used in this study included biological males and females, with no sex differences between the groups. We recruited patients with VKH from the Zhongshan Ophthalmic Center and Sun Yat-sen University, and we recruited those diagnosed based on clinical manifestations, coherent optical tomography, and indocyanine green fluorescein angiography, in line with the VKH’s Revised Diagnostic Criteria (70). All patients with VKH were newly diagnosed and free from prior treatment, with exclusion criteria covering coexisting conditions such as diabetes, hypertension, cancer, and other systemic disorders. Blood specimens were collected from 6 patients with VKH and 6 HCs for scRNA-Seq analysis, with no discernible differences in sex or age between the groups. All protocols received approval from the Medical Ethics Committee of the Guangzhou Zhongshan Ophthalmic Center (ID:2020KYPJ124).

### EAU model establishment.

The EAU model was induced s.c. using human IRBP_1-20_ (2 mg/mL; GL Biochem) emulsified in complete Freund’s adjuvant (Difco) that contained 2.5 mg of *M. tuberculosis* strain H37Ra (Difco; 1:1 v/v). Additionally, on days 0 and 2, pertussis toxin (0.25 μg) was i.p. administered (70).

On day 14 following immunization, mice were subjected to fundus examination and scored to assess EAU severity. Clinical scoring, ranging from 0 to 4 (71), was based on observable retinal vasculitis as well as choroidal and retinal infiltration/lesions (Supplemental Table 1); analyses of these tissues were conducted in a blinded manner.

### H&E staining.

Mouse eyes first underwent fixation and dehydration; then, they were paraffin embedded and sliced into 4 μm–thick sections for staining with H&E. Pathological scoring was conducted in a blinded manner according to the scoring criteria (71).

### Treatment protocols.

Mice were i.p. administered with a DGAT1 inhibitor, T863 (*n* = 6; 30 mg/kg; MCE), or an equivalent volume of vehicle control (*n* = 6; 0.1% DMSO/30% PEG-300/0.5% Tween80; Beyotime), daily for a continuous period of 14 days after immunization. In the in vitro experiments, a concentration of 20 μM T863 was employed.

### PBMC treatment.

PBMC were stimulated with CD3/28 Dynabeads (Thermo Fisher Scientific) alone or with additional T863 (20 μM). After 72 hours of incubation, they were prepared for further analysis.

### CDLN cell isolation and treatment.

Cells were isolated from CDLNs and retina of mice for in vivo detection. CDLN cells were cultured with IRBP_1-20_ (20 μg/mL) and supplemented with extra T863 (20 μM) for 72 hours, using for further analysis.

### Adoptive transfer experiment.

The CDLN cells from EAU mice (day 14) were cultured with IRBP_1-20_ (20 μg/mL), with or without T863, for a duration of 72 hours. Subsequently, Th17 cells were sorted (1 × 10^7^ living cells/mouse) and injected into WT mice (*n* = 6) via tail vein injection.

### Adenovirus transfection.

The adenoviruses used for DGAT1 knockdown or overexpression were procured from Hanheng Biotechnology. Cells were transduced with viral supernatants in the presence of 8 μg/mL polybrene (Beyotime) and subsequently cultured in complete medium comprising 45 mL 1640 medium, 5 mL FBS, 5 μL nonessential amino acids, 10 mM sodium pyruvate, and 50 μL of 50 mM β-mercaptoethanol (Thermo Fisher Scientific). Fluorescence signals became observable within 48–72 hours.

### Flow cytometry.

Following live/dead staining, harvested mouse cells underwent surface marker staining. Mouse samples were stained with PerCP/Cyanine5.5 anti-CD4 (BioLegend, 100434, 0.2 μg/mL), PE/Cyanine7 anti-CD25 (BioLegend, 102016, 0.2 μg/mL), and PE anti-CD69 (BioLegend, 104507, 0.2 μg/mL). Human samples were stained with PE/Cyanine7 anti-CD3 (BioLegend, 300419, 0.2 μg/mL), PerCP /Cyanine5.5 anti-CD4 (BioLegend, 317428, 0.2 μg/mL), and PerCP /Cyanine5.5 anti-CD8a (BioLegend, 300924, 0.2 μg/mL).

For intracellular markers, mouse cells were incubated with ionomycin (500 ng/mL, Sigma-Aldrich), brefeldin A (1 μg/mL, Sigma-Aldrich), and phorbolmyristate acetate (5 ng/mL, Sigma-Aldrich) for 5 hours. After fixation and permeabilization, the cells were stained with Alexa Fluor 647 anti–IL-17A (BioLegend, 506912, 0.2 μg/mL), PE anti–IFN-γ (BioLegend, 505808, 0.2 μg/mL), anti-DGAT1 (Bioss, BB11214840, 0.2 μg/mL), FITC anti-Foxp3 (Invitrogen, 11-5773-82, 0.2 μg/mL), Alexa Fluor 647 anti-Ki67 (BioLegend, 652407, 0.2 μg/mL), FITC-BODIPY FL C_16_ (Invitrogen, D3821, 25ng/mL), FITC-BODIPY 493/503 (Invitrogen, D3922, 200 ng/mL), FITC-BODIPY 581/591 C11 (Invitrogen, D3861, 5μM), Txnip (ProteinTech, 18243-1-AP), and anti–rabbit IgG (H + L), F(ab’)_2_ Fragment (Alexa Fluor 647 conjugate) secondary antibody (Cell Signaling Technology, 4414, 1:1,000). In the case of intracellular staining with human samples, the collected cells underwent fixation, permeabilization, and staining with Alexa Fluor 647 anti–IL-17A (BioLegend, 512309, 0.2 μg/mL), PE anti-DGAT1 (Bioss, BB11214840, 0.2 μg/mL), FITC BODIPY 581/591 C11 (Invitrogen, D3861, 5μM). Subsequently, flow cytometric analysis was performed using the BD LSRFortessa flow cytometer (BD Biosciences), and the data were analyzed using FlowJo software version 10.0.7 (BD Biosciences).

### scRNA-Seq data processing.

The scRNA-Seq libraries were prepared following the manufacturer’s instructions, with minor modifications, using the Chromium Single Cell 5′ Library and Gel Bead Kit (10X Genomics, 120237). Reverse transcription, emulsion breaking, barcoded-cDNA purification using Dynabeads, and PCR amplification were sequentially performed. The amplified cDNA was used for constructing the 5′ gene expression library. Fragmentation and end-repair, double-size selection with SPRIselect beads as well as sequencing were conducted on 50 ng of amplified cDNA using the NovaSeq platform (Illumina NovaSeq6000) to generate paired-end reads. Initial data processing was carried out using CellRanger software v7.1.0 (10X Genomics). Integration and clustering of the data were performed using the Seurat package (v4.3.0) in R (v4.1.3). The influence of batch effect across different samples was rectified using the Harmony package (v1.0) in R (72).

### Isolation of CDLNs for scRNA-Seq.

The CDLNs were collected from 3 mice per group and pooled to create a unified sample for single-cell library construction. Following homogenization, the pooled cells were cultured in RPMI-1640 medium (Thermo Fisher Scientific) supplemented with 2% FBS (Thermo Fisher Scientific), 3 mg/mL Collagenase IV (Sigma-Aldrich), penicillin-streptomycin (Pen/Strep) antibiotics (Thermo Fisher Scientific), and 40 mg/mL DNase I (Sigma-Aldrich). The mixture was then incubated at 37°C for 15 minutes. Subsequently, the digested cells were harvested and sieved through a 70 mm cell strainer. The resulting single-cell suspension attained a concentration of 1 × 107 cells/mL (with a viability of ≥ 85%), as confirmed using the Countess® II Automated Cell Counter.

### Isolation of PBMCs for scRNA-Seq.

Peripheral blood samples were collected from HCs and patients with VKH for pipeline assessment. Subsequently, PBMCs were isolated using Ficoll-Hypaque solution (GE Healthcare) and subjected to standard density gradient centrifugation (400*g*). The viability of all samples exceeded 85%.

### Dimensionality reduction and clustering analysis.

For the scRNA-Seq data analysis, normalization was conducted employing the “NormalizeData” function within the Seurat package in R. Subsequently, we utilized the “FindClusters” function to group cells and employed the “RunUMAP” function to visualize the data using a 2-dimensional UMAP algorithm. Furthermore, we utilized the “FindAllMarkers” function to pinpoint marker genes for distinct clusters.

### DEG analysis.

We conducted DEG analysis across different cell types among various groups using the “FindMarkers” function (adjusted *P* < 0.05, |log_2_FC| > 0.25). Prior to DEG analysis, cell types with either no representation or fewer than 3 cells in the compared groups were excluded.

### Gene funtional analysis.

Gene ontology (GO) and pathway enrichment analysis was conducted utilizing the Metascape webtool (www.metascape.org) (73). The *P* values of GO terms are determined through the cumulative hypergeometric distribution provided by the Metascape webtool. Pathways associated with diseases were visually represented using the pheatmap (v1.0.12) in R.

### Cell viability assay.

Cell viability was assessed using a CCK-8 assay kit (Dojindo Laboratories) following the manufacturer’s protocol. Specifically, cells were treated with or without T863 for 72 hours. After incubating with CCK-8 solution for 3 hours at 37°C, the absorbance of each well was measured at 450 nm using an automated ELISA reader.

### Single-cell flux estimation analysis (scFEA).

The single-cell Flux Balance Analysis (scFBA) method was utilized to assess metabolite levels using scRNA-Seq data (74). In Python, metabolic fluxomes and abundances were computed for specific cell types using scFBA. Following the computation of metabolic flux and metabolite levels, selected metabolic modules and all metabolites were visualized using a ridge plot.

### Protein-protein interaction network construction and hub gene prediction.

Using upregulated DEGs as a basis, we utilized String (75) to establish the protein-protein interaction (PPI) network, which was then visualized through Cytoscape (v3.9.1). Subsequently, the hub genes were identified using the CytoHubba plugin, employing 8 distinct algorithms (76).

### Data availability statement.

The scRNA-Seq data of T863-treated mice and human PBMC in this paper were deposited in the Genome Sequence Archive at the BIG Data Center, Beijing Institute of Genomics (BIG; https://bigd.big.ac.cn/gsa/), Chinese Academy of Sciences (GSA accession no.: CRA020249 [mice], HRA007222 [human]). Data supporting this study will be made available upon reasonable request.

### Statistics.

Data analysis and visualization were conducted using GraphPad Prism Software v 8.0.2. The results are presented as the mean ± SD. Statistical analysis was carried out employing an unpaired 2-tailed Student’s *t* test, 1-way ANOVA. We conducted DEG analysis across different cell types among various groups using the “FindMarkers” function with Wilcoxon rank sum test (adjusted P < 0.05, |log2FC| > 0.25). Prior to DEG analysis, cell types with either no representation or fewer than 3 cells in the compared groups were excluded. *P* < 0.05 were considered to be statistically significant. 

### Study approval.

IACUC approval (Zhongshan Ophthalmic Center, Sun Yat-Sen University) was obtained for all animal experiments. All protocols received approval from the Medical Ethics Committee of the Guangzhou Zhongshan Ophthalmic Center (ID: 2020KYPJ124). Written informed consent was diligently obtained from all human participants. Adhering strictly to the animal welfare and usage guidelines, the mice were accommodated in a specific pathogen-free condition. Our study adhered to the Declaration of Helsinki guidelines, and written informed consent was obtained from all participants.

### Data availability.

scRNA-Seq data related to immunosuppressive agents are available from Genome Sequence Archive at the BIG Data Center (BIG; https://bigd.big.ac.cn/gsa/) and Chinese Academy of Sciences (GSA accession no. CRA006097 for cyclosporine-related data, CRA014816 for dimethyl fumarate–related data, and CRA009662 for mycophenolate mofetil–related data). Values for all data points in graphs are reported in the Supporting Data Values file.

## Author contributions

WS and LF designed research; TW, RD, QJ, and ZL performed research; BZ, RD, LJ, TW, JL, and ZL analyzed data; and TW and BZ wrote the paper, revised by LF. All authors have read and approved the final manuscript.

## Supplementary Material

Supplemental data

Supporting data values

## Version 1 04/08/2025

Electronic publication
