# Supplementary material for: Lipid metabolism analysis reveals that DGAT1 regulates Th17 survival by controlling lipid peroxidation in uveitis
Source: JCI Insight. 2025 Apr 8;10(7):e184072. doi: 10.1172/jci.insight.184072 (PMC11981632; doi:10.1172/jci.insight.184072)
Supplement: Supplemental data [file jciinsight-10-184072-s116.pdf]

Supporting Information for

Lipid metabolism analysis reveals that DGAT1 regulates Th17 survival by controlling lipid peroxidation in uveitis

Tianfu Wang[1, #], Runping Duan[1, #], Zhaohuai Li[1, #], Bowen Zhang[2, #], Qi Jiang, Loujing Jiang, Jianjie Lv, Yingqi Li[3, *], Wenru Su[1, *], Lei Feng[3, *]

[1] State Key Laboratory of Ophthalmology, Zhongshan Ophthalmic Center, Sun Yat-sen University, Guangdong Provincial Key Laboratory of Ophthalmology and Visual Science, Guangzhou 510060, China
[2] Department of Clinical Medicine, Zhongshan School of Medicine, Sun Yat-Sen University, Guangzhou 510275, China.
[3] Eye center, The Second Affiliated Hospital, Zhejiang University School of Medicine, Hangzhou, 310009, China

[#] These authors contributed equally

[*]Correspondence: Wenru Su, suwr3@mail.sysu.edu.cn. Lei Feng, leifeng@zju.edu.cn.

This file includes:

Figures S1 to S17
Clinical scoring criteria of EAU and pathological scoring criteria of EAU
Abbreviations
Information of HC and VKH sample

Figure. S1

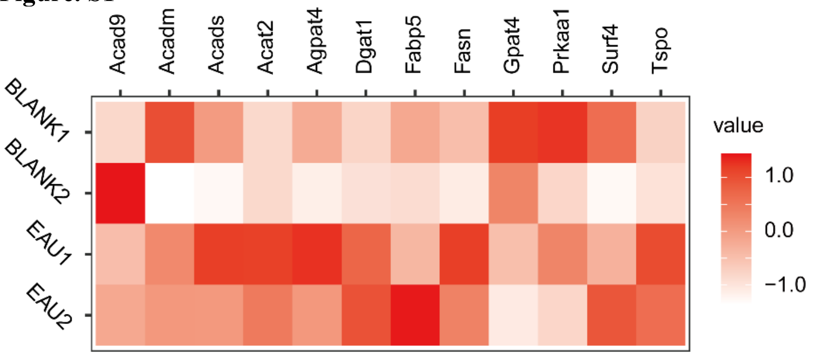

Figure.S 1 Heatmap showing lipid metabolism related genes of Th1 cells in the EAU and the control groups.

EAU: experimental autoimmune uveitis.

Figure. S2

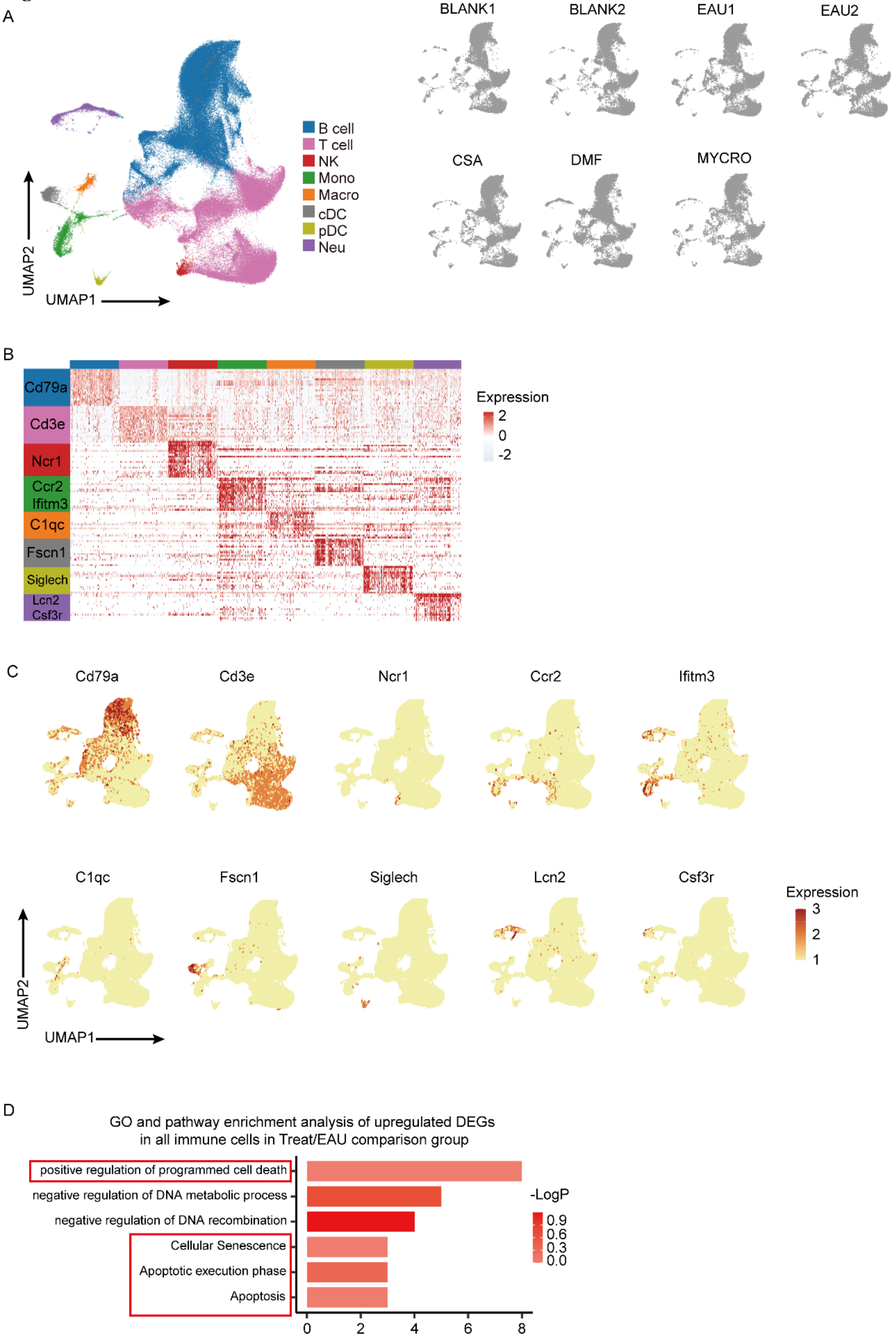
A
BLANK1
BLANK2
EAU1
EAU2
CSA
DMF
MYCRO
B cell
T cell
NK
Mono
Macro
cDC
pDC
Neu
UMAP2
UMAP1
B
Cd79a
Cd3e
Ncr1
Ccr2
Ifitm3
C1qc
Fscn1
Siglech
Lcn2
Csf3r
Expression
2
0
-2
C
Cd79a
Cd3e
Ncr1
Ccr2
Ifitm3
C1qc
Fscn1
Siglech
Lcn2
Csf3r
Expression
3
2
1
UMAP2
UMAP1
D
GO and pathway enrichment analysis of upregulated DEGs
in all immune cells in Treat/EAU comparison group
positive regulation of programmed cell death
negative regulation of DNA metabolic process
negative regulation of DNA recombination
Cellular Senescence
Apoptotic execution phase
Apoptosis
-LogP
0.9
0.6
0.3
0.0
0
2
4
6
8

Figure. S 2 Clusters of major immune cell populations and GO analysis of upregulated DEGs in TRE/EAU group (A) UMAP plot showing clusters of T cells from all mice groups. (B) Heatmap showing scaled expression of discriminative gene sets for major immune cell types in CDLN cells from all mice groups. (C) UMAP plots of canonical markers for immune cell subsets. (D) Representative GO terms and KEGG pathways enriched in upregulated DEGs of all immune cells in TRE/EAU comparison group.

Figure. S3

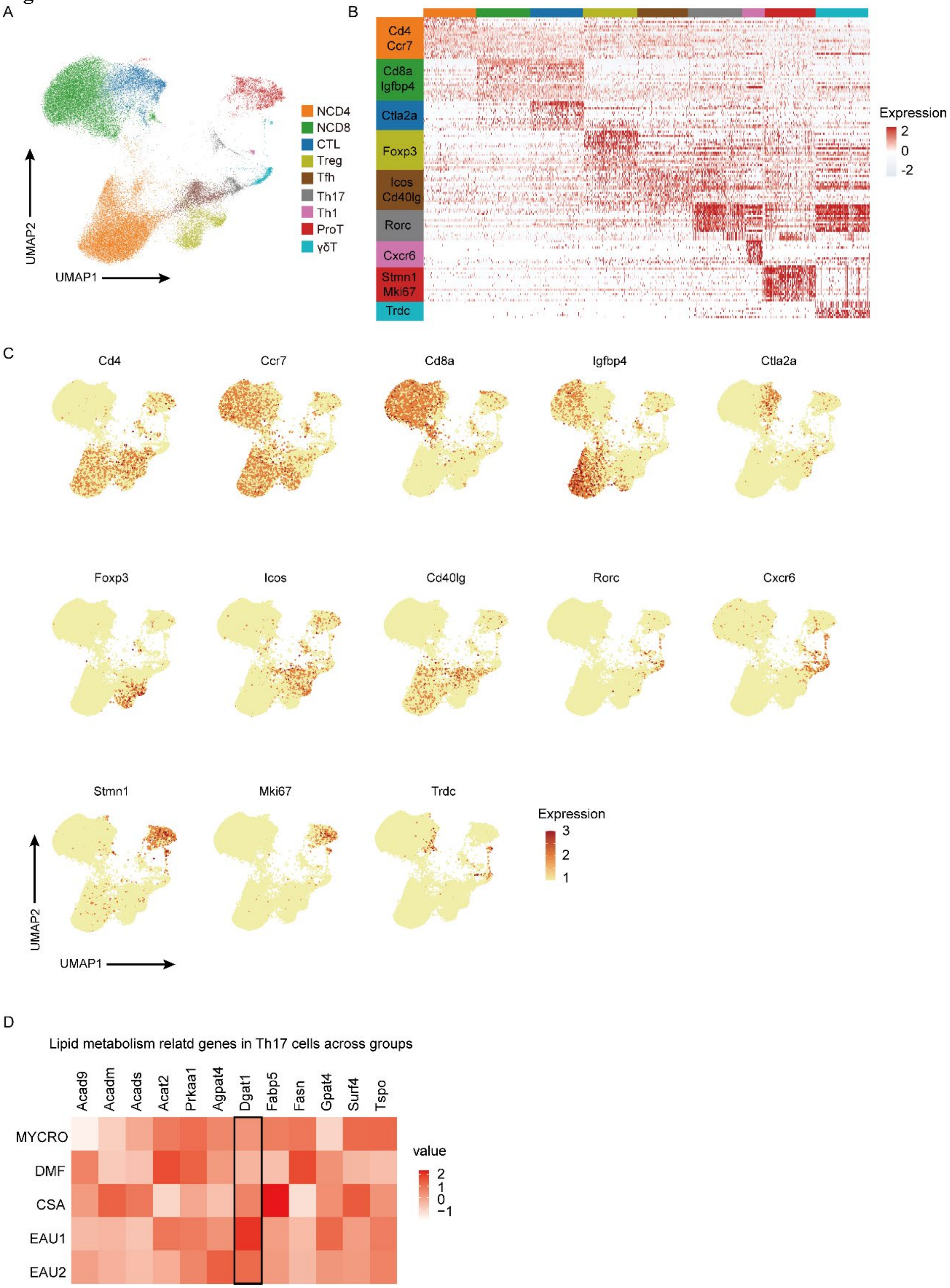

Figure.S 3 Clusters of T cell subtypes populations in EAU and TRE groups (A) UMAP plot showing clusters of T cells from all mice groups. (B) Heatmap showing scaled expression of discriminative gene sets for major immune cell types in T cells. (C) UMAP plots of canonical markers for T cell subsets. (D) Heatmap showing expression levels of lipid metabolism related genes.

Figure. S4

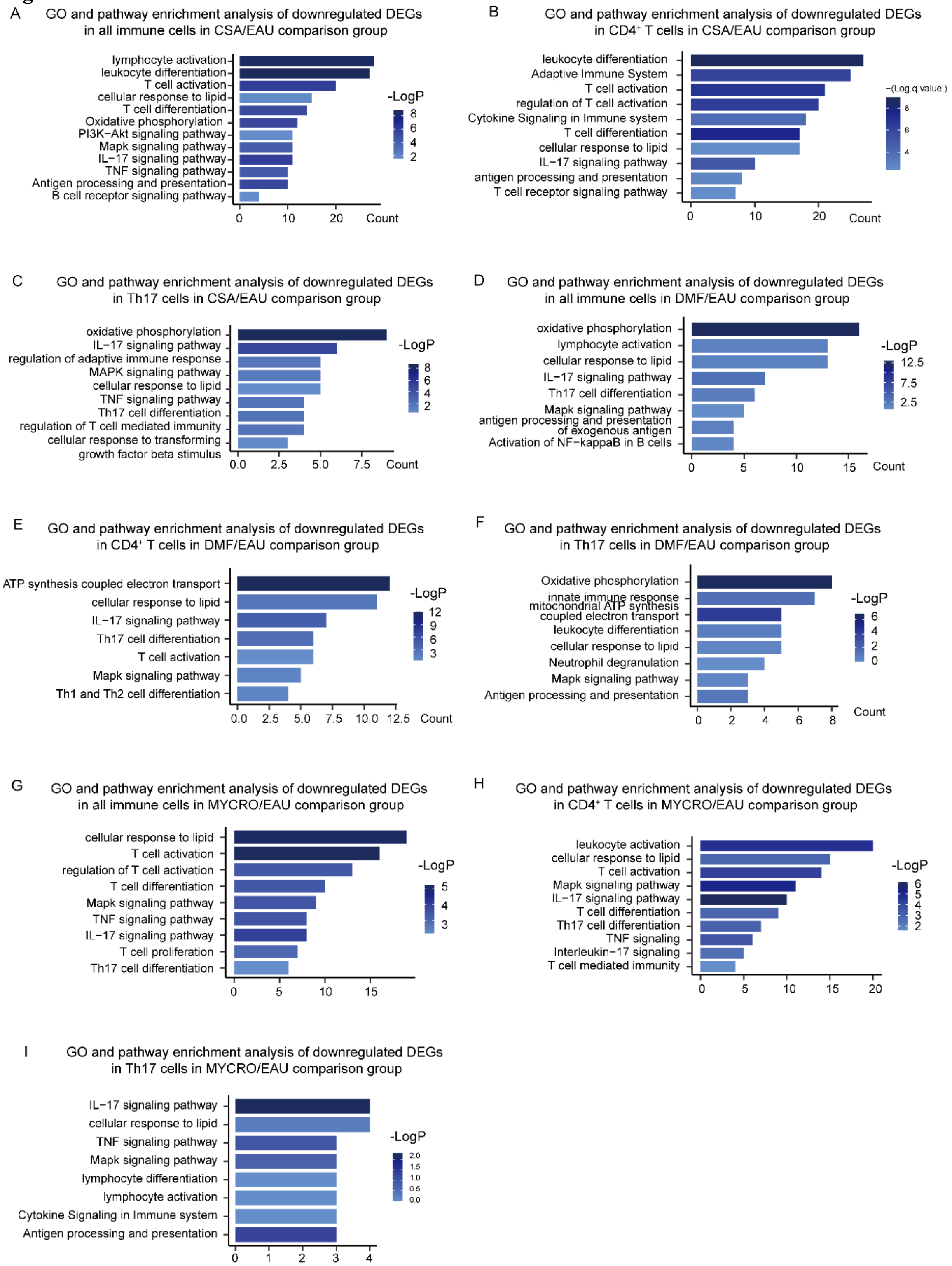

Figure.S 4 (A-C) Representative GO terms and KEGG pathways enriched in downregulated DEGs of total immune cells (A), CD4+T cells (B) and Th17 cells (C) in the CSA/EAU comparison group. (D-F) Representative GO terms and KEGG pathways enriched in downregulated DEGs of total immune cells (D), CD4+T cells (E) and Th17 cells (F) in the DMF/EAU comparison group. (G-I) Representative GO terms and

KEGG pathways enriched in downregulated DEGs of total immune cells (G), CD4+T cells (H) and Th17 cells (I) in the CSA/EAU comparison group.
EAU: experimental autoimmune uveitis. MYCRO: mycophenolate Mofetil, DMF: dimethyl Fumarate, CSA: cyclosporine.

Figure. S5

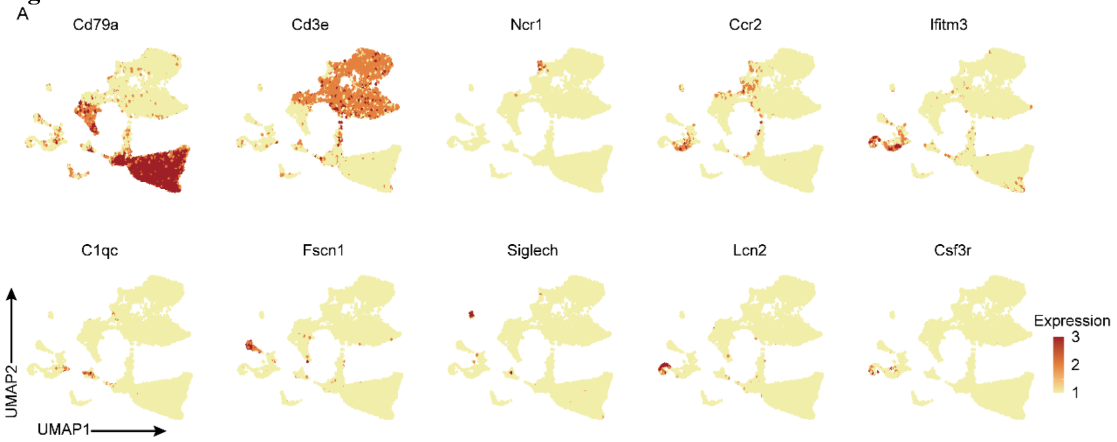

Figure.S 5 (A) UMAP plots of canonical markers for immune cell subsets from T863 group.

Figure. S6

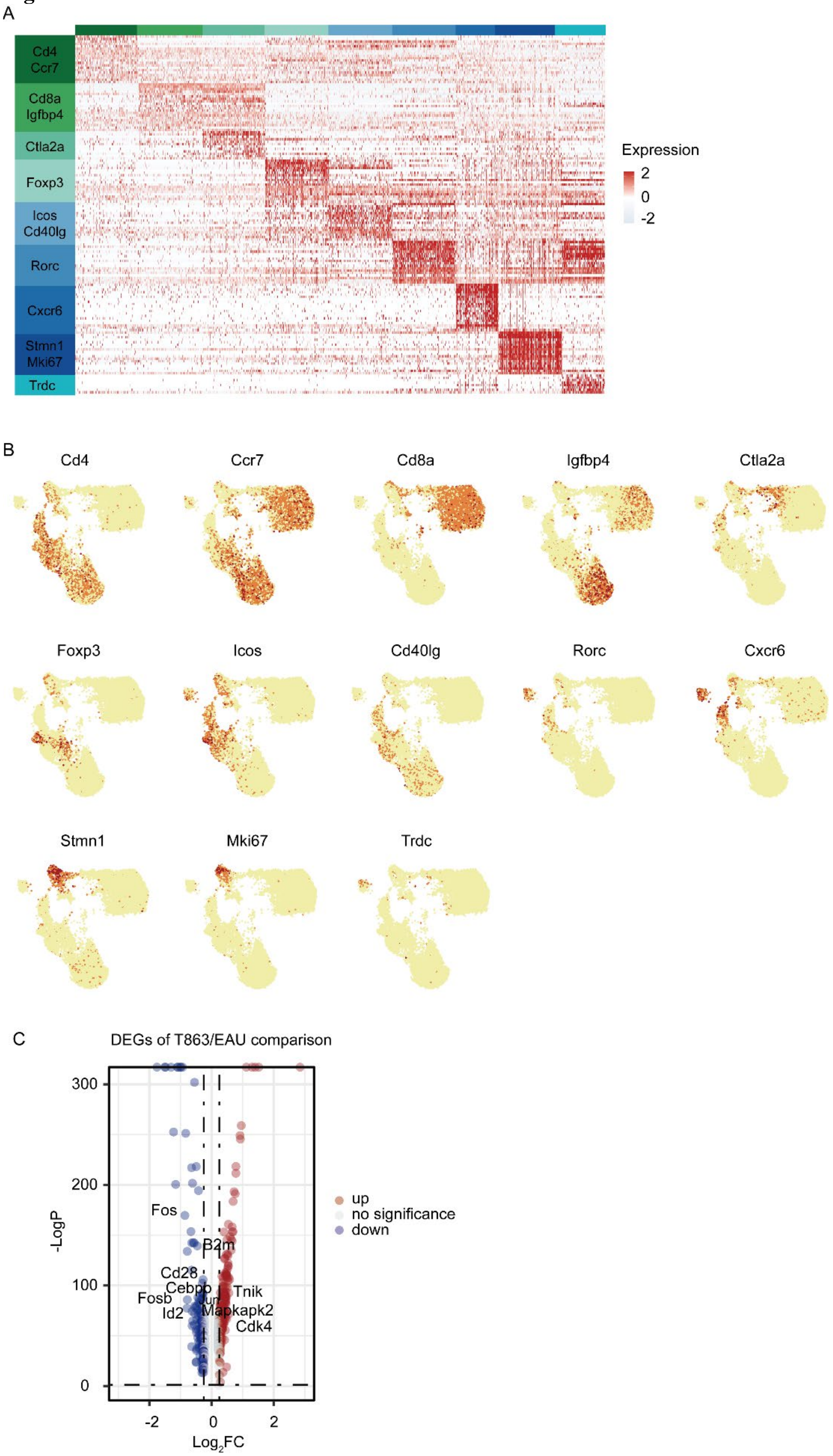
A
Cd4
Ccr7
Cd8a
Igfbp4
Ctla2a
Foxp3
Icos
Cd40lg
Rorc
Cxcr6
Stmn1
Mki67
Trdc
Expression
2
0
-2
B
Cd4
Ccr7
Cd8a
Igfbp4
Ctla2a
Foxp3
Icos
Cd40lg
Rorc
Cxcr6
Stmn1
Mki67
Trdc
C
DEGs of T863/EAU comparison
-LogP
Log2FC
Fos
B2m
Cd28
Cebpb
Fosb
Jun
Id2
Tnik
Mapkapk2
Cdk4
up
no significance
down

Figure. S 6 Clusters of T cell subtypes populations in EAU and T863 groups and Volcano plot showing DEGs of total immune cells. (A) Heatmap showing scaled expression of discriminative gene sets for major immune cell types in T cells from T863 group. (B) UMAP plots of canonical markers for T cell subsets from T863 group. (C) Volcano plot showing upregulated and downregulated DEGs of total immune cells in the T863/EAU comparison groups. Red and blue dots indicate upregulated and downregulated DEGs in T863 groups compared to EAU groups, respectively. Significance was determined using "FindMarkers" functions of Seurat package with Wilcoxon Rank Sum test and adjusted by Bonferroni correction.

Figure. S7

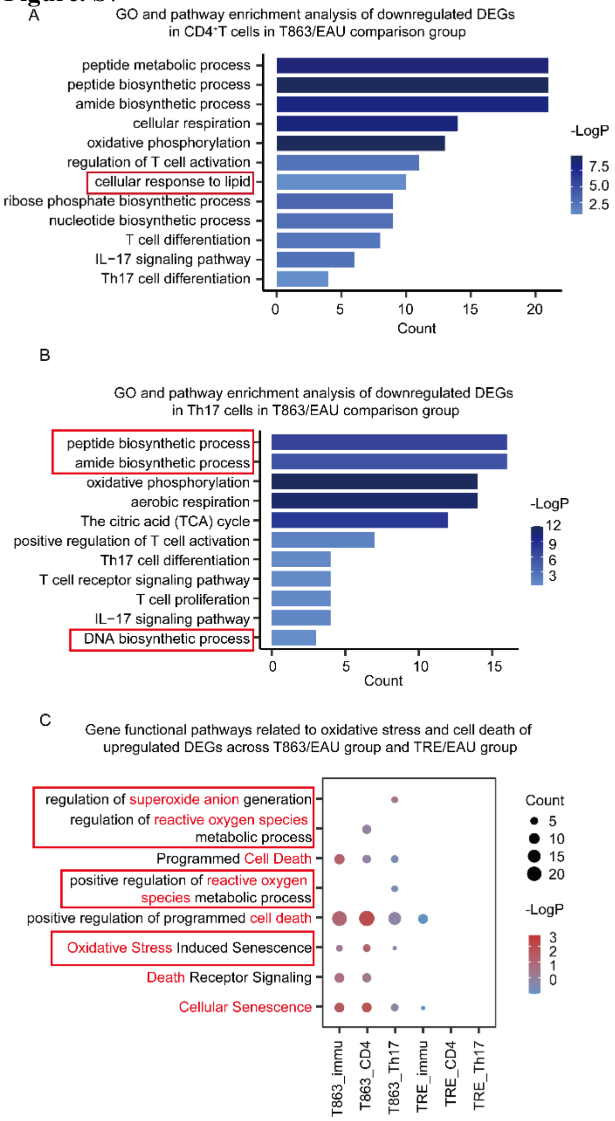

Figure. S 7 Representative GO terms and KEGG pathways in T863/EAU group and a comparison between T863/EAU group and TRE/EAU group. (A) Representative GO terms and KEGG pathways enriched in downregulated DEGs of CD4^{+} cells in the T863/EAU comparison group. (B) Representative GO terms and KEGG pathways enriched in downregulated DEGs of Th17 cells in the T863/EAU comparison group. (C) Functional enrichment analysis showing the GO and pathways involving increased oxidative stress and cell death in T863/EAU group and TRE/EAU group.

Figure. S8

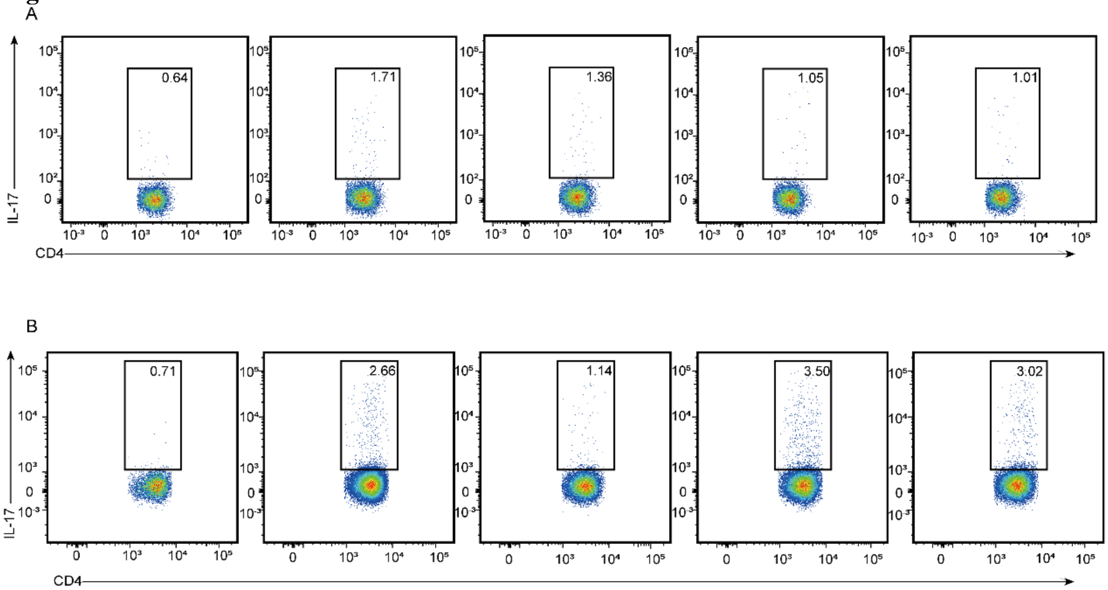

Figure. S 8 Representative flow cytometry chart of in vitro experiments exploring the effects of T863 and FER1 on the proportion of Th17 cells. (A) CD4$^+$T cells of CDLN cells from EAU mice cultured with no IRBP$_{1-20}$, IRBP$_{1-20}$ alone or with IRBP$_{1-20}$ with escalating doses of T863(0-20μM) for 72h. Flow cytometry showed the proportion of IL-17A$^+$ cells. (B) CD4$^+$T cells of CDLN cells from EAU mice cultured with/without IRBP, with/without T863, with/without FER1 for 72h.Proportions of CD4$^+$IL-17A$^+$ cells were measured by flow cytometry.

Figure. S9

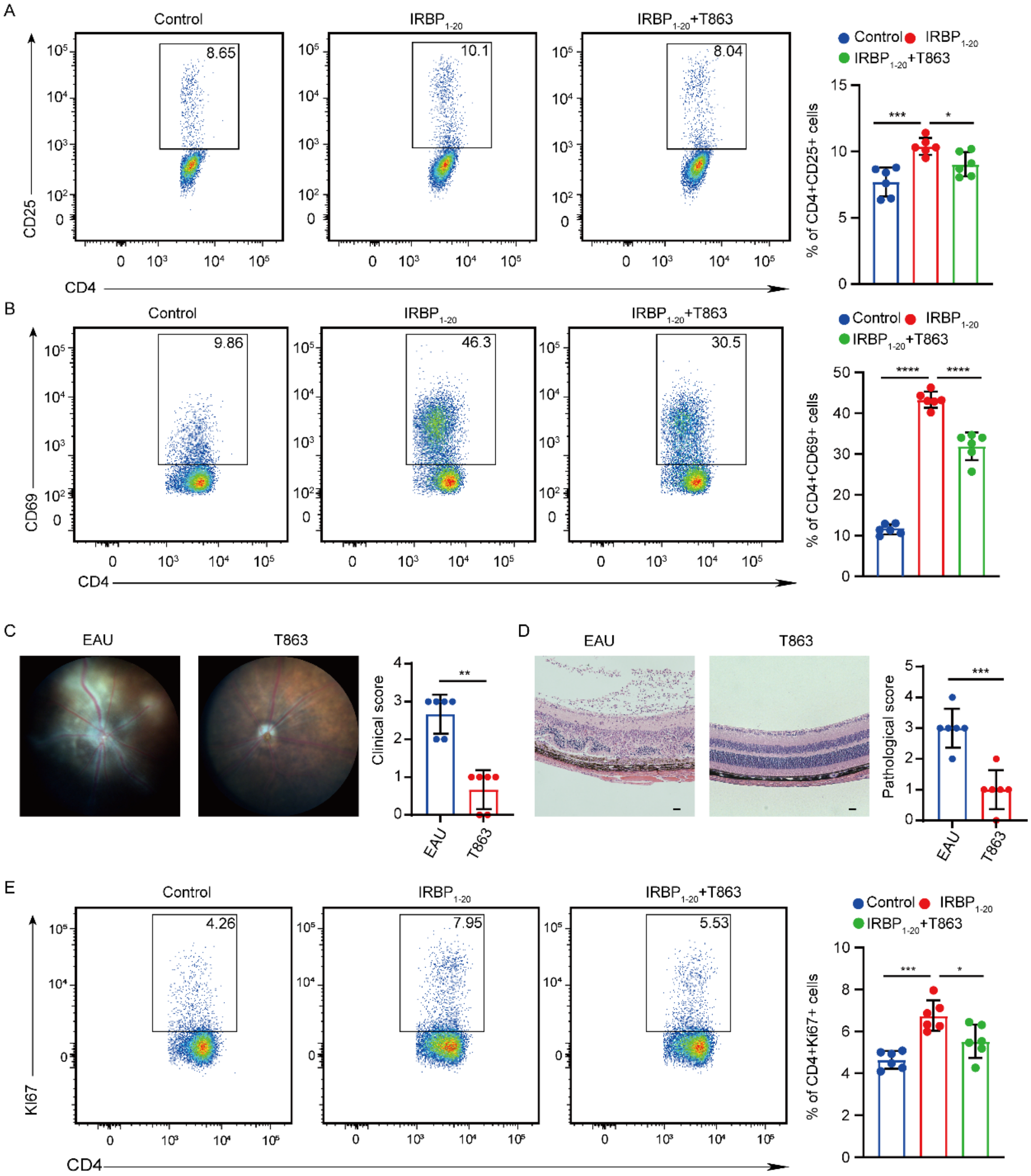

Figure. S 9 Effect of T863 on CD4⁺ T cell active phenotype and proliferation phenotype and adoptive experimental results. (A) $CD4^+$T cells of CDLN cells from EAU mice cultured with no $IRBP_{1\text{-}20}$, $IRBP_{1\text{-}20}$ alone or with $IRBP_{1\text{-}20}$ with T863(20μM) for 72h. Flow cytometry showed the proportion of $CD25^+$ cells. (n=6, data are presented as mean±SD. significance was determined using one-way ANOVA). (B) $CD4^+$T of CDLN cells cells from EAU mice cultured with no $IRBP_{1\text{-}20}$, $IRBP_{1\text{-}20}$ alone or with $IRBP_{1\text{-}20}$ with T863(20μM) for 72h. Flow cytometry showed the proportion of $CD69^+$ cells. (n=6, data are presented as mean±SD. significance was determined using one-way ANOVA). (C) Representative fundus images and clinical scores of eyes from the EAU mice and T863 treated mice (n=6, data are presented as mean±SD, significance was determined using unpaired two-tailed student's t test). (D) Representative histopathological images (hematoxylin and eosin staining) and pathological scores of eyes from the EAU mice and T863 treated mice (n=6, data are presented as mean±SD, significance was determined using unpaired two-tailed student's t test). Scale bars, 20μm. (E) CD4+T cells of CDLN cells from EAU mice cultured with no IRBP1-20, IRBP1-

20 alone or with $IRBP_{1-20}$ with T863(20μM) for 72h. Flow cytometry showed the proportion of $KI67^{+}$ cells. (n=6, data are presented as mean ± SD. significance was determined using one-way ANOVA). ns: no significance, *: $P < 0.05$, **: $P < 0.01$, ***: $P < 0.001$, ****: $P < 0.0001$. VKH: Vogt-Koyanagi-Harada Disease, HC: healthy controls.

Figure.S10

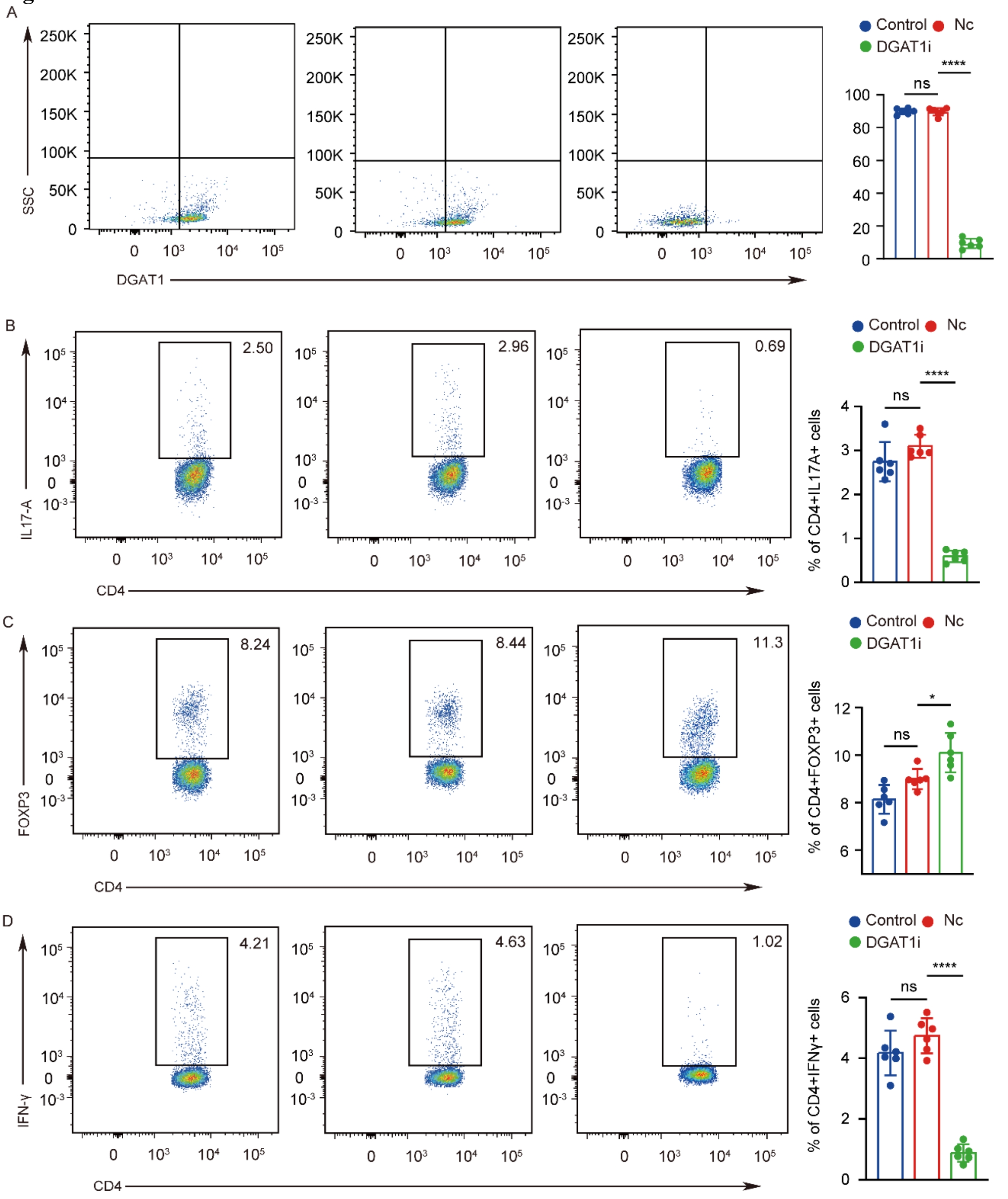

Figure. S10 DGAT1 knockdown regulated Th17/Treg balance (A) After treated with adenovirus or negative control (Nc), flow cytometry was performed to show the proportion of DGAT1+ cells in total $CD4^+$ cells. Each group contains six mice. (B-D) After treated with adenovirus or negative control (Nc), CDLN cells from EAU mice were cultured with $IRBP_{1-20}$. Flow cytometry was performed to show the proportion of Th17 cells (B), Treg cells (C) and Th1 cells (D). ns: no significance, *: $P < 0.05$, ****: $P < 0.0001$. Control: cells for blank control, Nc: cells treated with negative control adenovirus, DGAT1i: cells treated with adenovirus for DGAT1 knockdown.

Figure. S11

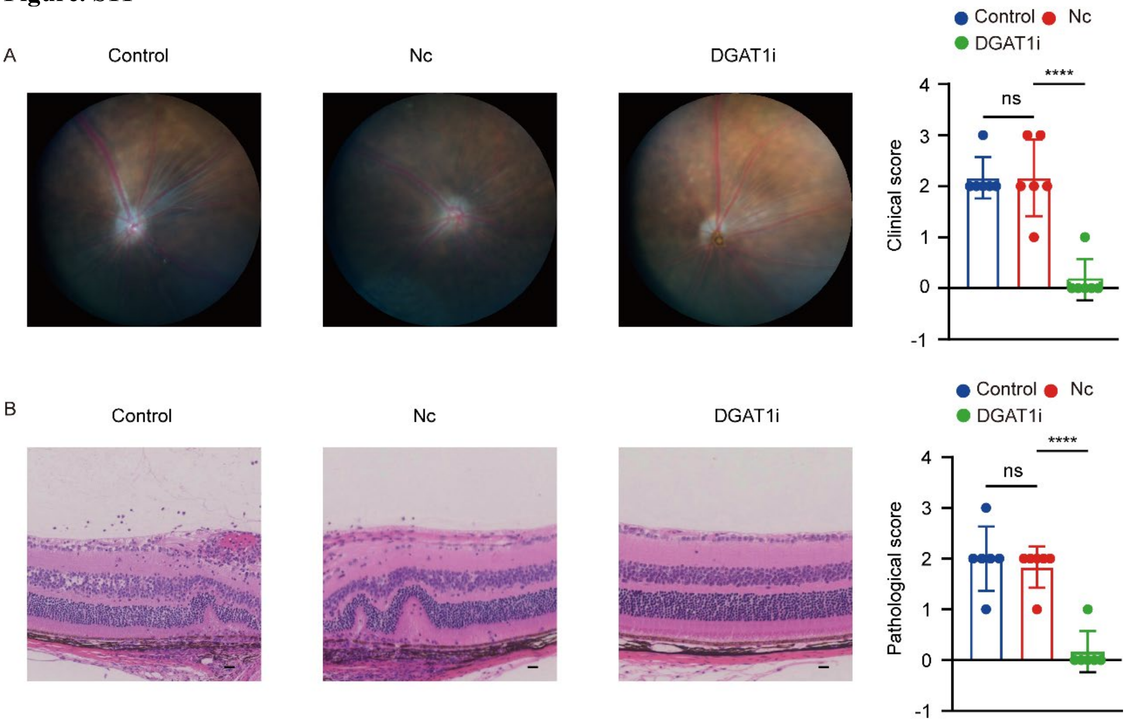

Figure. S 11 DGAT1 knockdown CD4^{+}T cells prevented the induction of EAU (A) Representative fundus images and clinical score after induction of DGAT1 knockdown CD4^{+} T cells cultured with IRBP$_{1\text{-}20}$ at day 14. (B) Representative histopathological images (hematoxylin and eosin staining) and pathological scores of eyes after induction of DGAT1 knockdown CD4^{+} T cells cultured with IRBP$_{1\text{-}20}$ at day 14. Scale bars, 20μm. ns: no significance, ****: $P < 0.0001$. Control: cells for blank control, Nc: cells treated with negative control adenovirus, DGAT1i: cells treated with adenovirus for DGAT1 knockdown.

Figure. S12

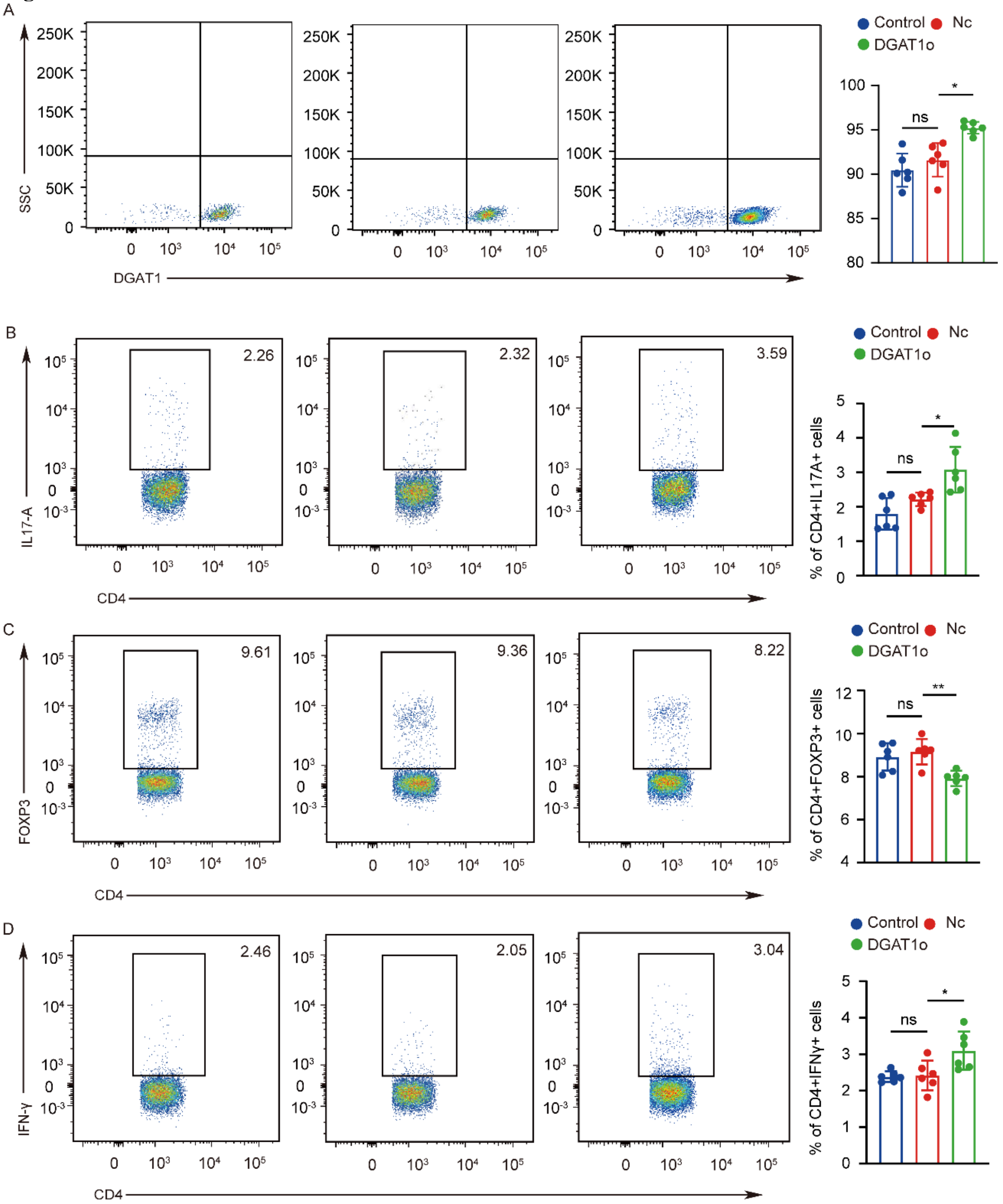

Figure. S 12 DGAT1 overexpression exacerbated Th17/Treg imbalance (A) After treated with adenovirus or negative control (Nc), flow cytometry was performed to show the proportion of DGAT1+ cells in total CD4^{+} cells. Each group contains six mice. (B-D) After treated with adenovirus or negative control (Nc), CDLN cells from EAU mice were cultured with IRBP$_{1\text{-}20}$. Flow cytometry was performed to show the proportion of Th17 cells (B), Treg cells (C) and Th1 cells (D). ns: no significance, *: $P < 0.05$, ****: $P < 0.0001$. Control: cells for blank control, Nc: cells treated with negative control adenovirus, DGAT1o: cells treated with adenovirus for DGAT1 overexpression.

Figure. S13

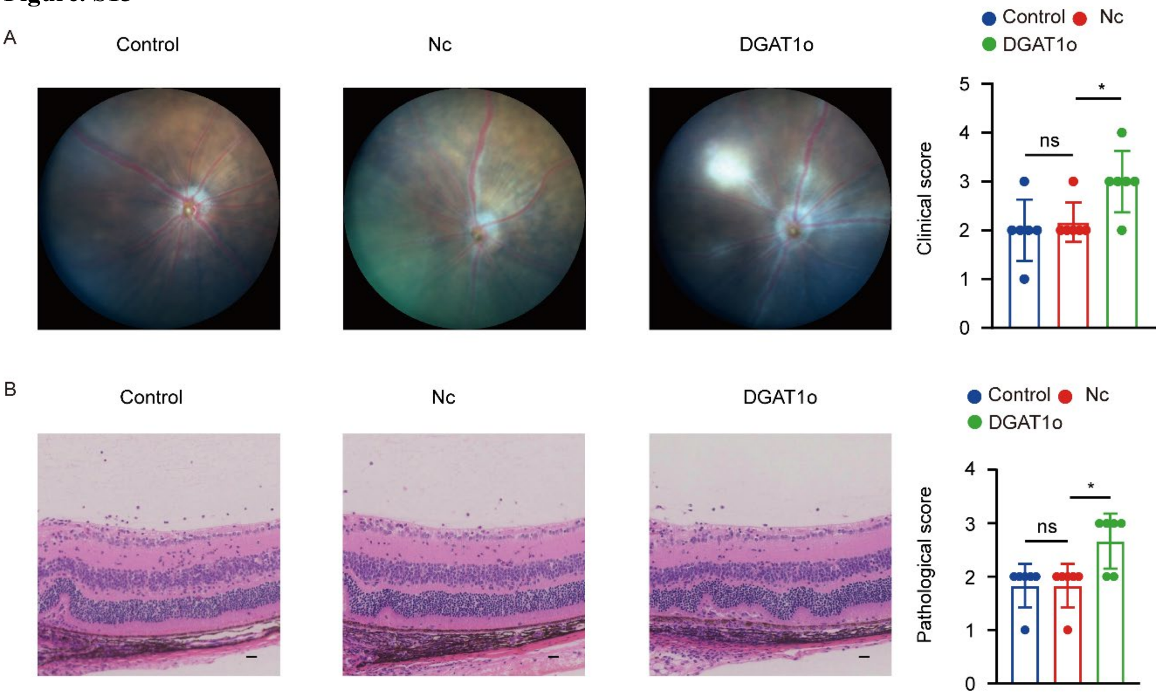

Figure. S 13 DGAT1 overexpression CD4^{+}T cells aggravated the induction of EAU (A) Representative fundus images and clinical score after induction of DGAT1 overexpression CD4^{+} T cells cultured with IRBP$_{1-20}$ at day 14. (B) Representative histopathological images (hematoxylin and eosin staining) and pathological scores of eyes after induction of DGAT1 overexpression CD4^{+} T cells cultured with IRBP$_{1-20}$ at day 14. Scale bars, 20μm. ns: no significance, ****: $P < 0.0001$. Control: cells for blank control, Nc: cells treated with negative control adenovirus, DGAT1o: cells treated with adenovirus for DGAT1 overexpression.

Figure. S14

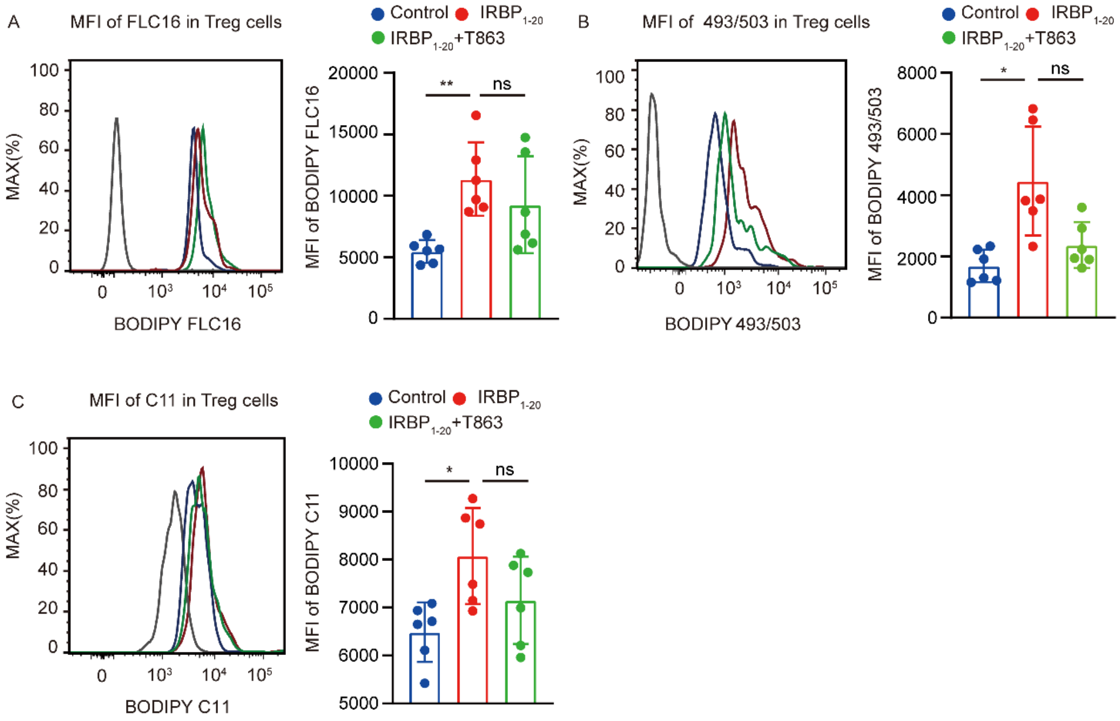

Figure. S 14 T863 induces changes in lipid metabolism in Treg cells (A-C) Representative histograms and MFI values of BODIPY FLC16 (A), BODIPY 493/503 (B) and BODIPY 581/591 C11 (C) in Treg cells from CDLN cells of EAU mice cultured with T863 for 72h(n=6, data are presented as mean ± SD, significance was determined using one-way ANOVA). ns: no significance, *: $P < 0.05$, **: $P < 0.01$.

Figure. S15

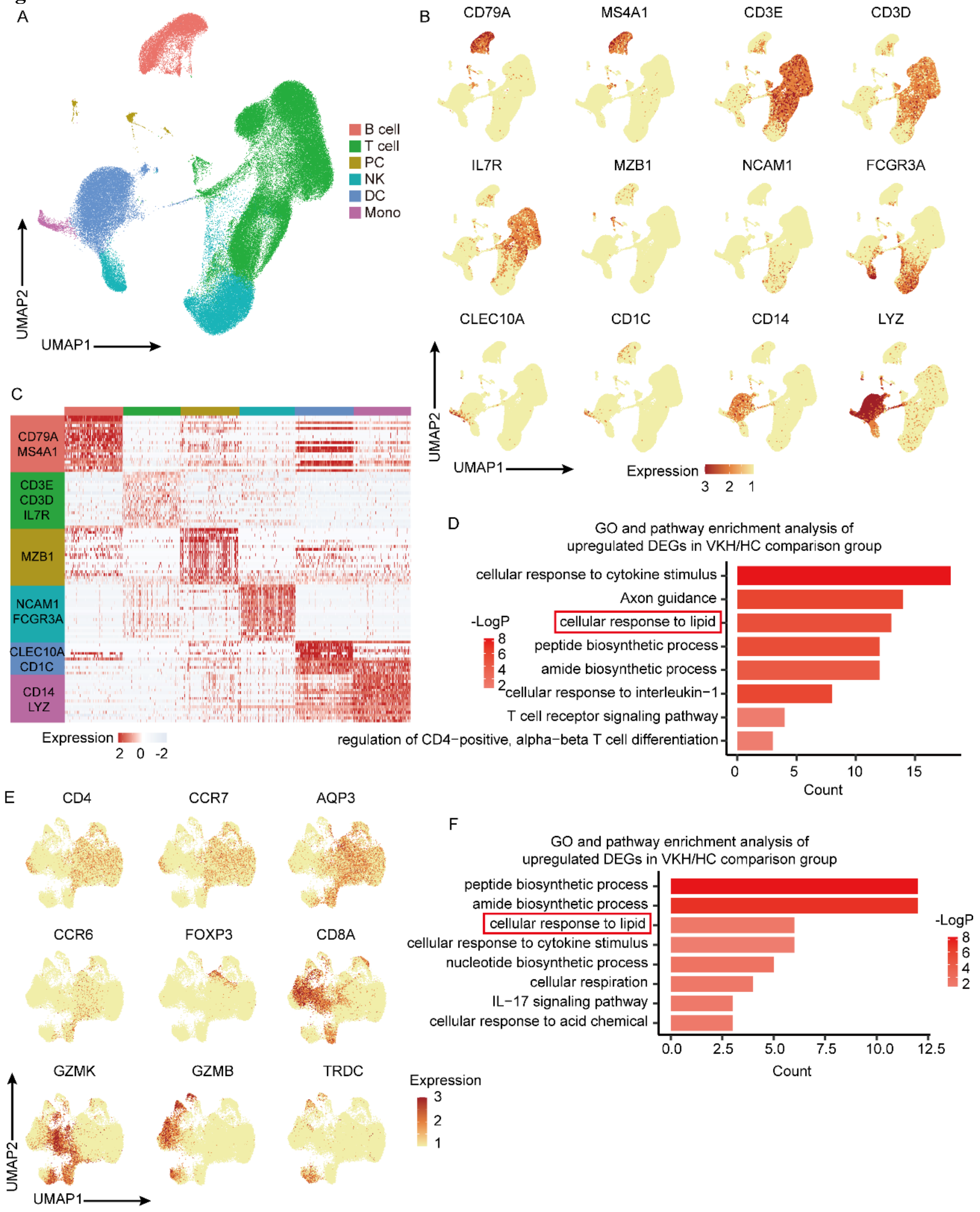

Figure. S 15 Clusters of major immune cells and T cell subtypes populations in VKH and HC groups and results of GO and pathway enrichment analysis of DEGs in VKH/HC group. (A) UMAP clustering of T cells from VKH group and HC group. (B) UMAP plots of canonical markers for immune cell subsets of VKH group and HC group. (C) Heatmap showing scaled expression of discriminative gene sets for major immune cell types in CDLN cells from VKH group and HC group. (D) Representative GO terms and KEGG pathways enriched in upregulated DEGs of all immune cells in the VKH/HC comparison group. (E) UMAP plots of canonical markers for T cell subsets VKH group and HC group. (F) Representative GO terms and KEGG pathways enriched in upregulated DEGs of T cells in the VKH/HC comparison group. VKH: Vogt-Koyanagi-Harada Disease, HC: healthy controls.

Figure. S16

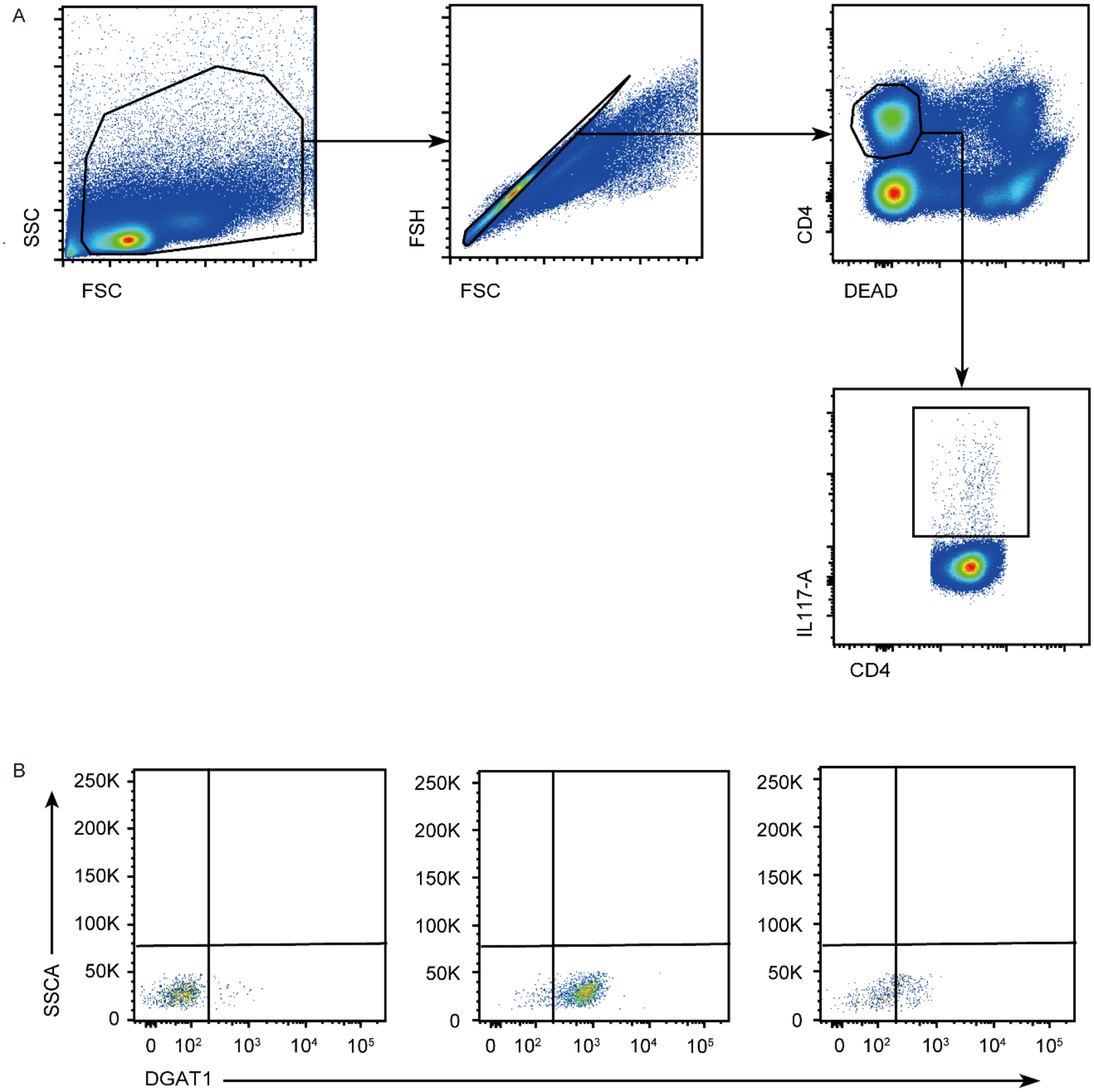

Figure. S 16 Gating strategy including live/dead staining for flow analysis and isotype figures of DGAT1 in Th17 cells. (A) Figures showing the gating strategy including live/dead staining for flow analysis. (B) The isotype figures of DGAT1 in Th17 cells.

Figure. S17

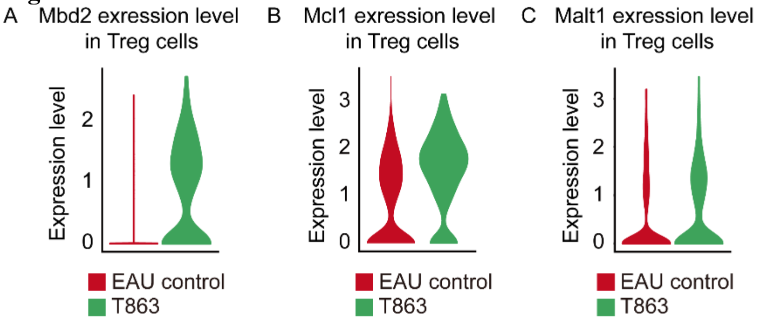

Figure.S 17 Violin plots of Mbd2 (A), Mcl1 (B) and Malt1 (C)in Th17 cell subsets from all mice groups.

Table S1 Clinical scoring criteria of EAU and pathological scoring criteria of EAU

**Clinical scoring criteria of EAU**	
**Grade**	**Criteria for fundoscopy**
0	Normal retina
0.5	Few (<3), small and focal lesions; minimal vasculitis and vitritis
1	Mild vasculitis; multiple, peripheral and focal lesions
2	Retinal edema; severe vasculitis (large size, thick wall, infiltrations); diffuse chorioretinal lesions and/or infiltrations; linear lesions
3	Retinal edema; pattern of linear lesions; large confluent chorioretinal lesions; subretinal hemorrhages
4	Large retinal detachmen
**pathological scoring criteria of EAU**	
**Grade**	**Criteria for histopathology**
0	Normal retina structure
0.5	Mild inflammatory cell infiltration. No tissue damage
1	Infiltration; retinal folds and focal retinal detachments; few small granulomas in choroid and retina, perivasculitis
2	Moderate infiltration; retinal folds, detachments and focal photoreceptor cell damage; small to medium sized granulomas, perivasculitis and vasculitis
3	Medium to heavy infiltration; extensive retinal folding with detachments, moderate photoreceptor cell damage; medium sized granulomatous lesions; subretinal neovascularization
4	Heavy infiltration; diffuse retinal detachment with serous exudate and subretinal bleeding; extensive photoreceptor cell damage; large granulomatous lesions; subretinal neovascularization

Abbreviations

CDLN	Cervical draining lymph nodes
scRNA-seq	Single-cell RNA sequencing
EAU	Experimental autoimmune uveitis
DGAT1	Diacylglycerol O-acyltransferase 1
VKH	Vogt-Koyanagi-Harada disease
FA	Fatty acid

LN	Lymph node
cDC	classical dendritic cell
pDC	plasmacytoid dendritic cell
Neu	neutrophil
Mono	monocyte
Macro	macrophage
DEG	differentially expressed gene
NCD4	naïve $CD4^+$ T cell
Treg	regulatory T cells
Th17	T helper 17 cell
ProT	proliferative T cell
Tfh	T follicular helper cell
γδT	γδT cell
CTL	$CD8^+$ T cells with cytotoxic activity
NCD8	naïve $CD8^+$ T cell

Information of HC and VKH sample

Group	Gender	Age	Single-cell RNAseq	Flow cytometry
VKH1	male	16	YES	NO
VKH2	female	61	YES	NO
VKH3	male	45	YES	NO
VKH4	female	39	YES	NO
VKH5	male	57	YES	NO
VKH6	male	34	YES	NO
VKH7	male	35	NO	YES
VKH8	male	46	NO	YES
VKH9	male	31	NO	YES
VKH10	male	24	NO	YES
VKH11	male	56	NO	YES
VKH12	female	45	NO	YES
VKH13	female	34	NO	YES
VKH14	female	47	NO	YES
VKH15	female	38	NO	YES
VKH16	female	46	NO	YES
HC1	male	20	YES	NO
HC2	female	56	YES	NO
HC3	male	39	YES	NO
HC4	female	41	YES	NO
HC5	male	55	YES	NO
HC6	male	32	YES	NO
HC7	male	26	NO	YES

HC8	male	36	NO	YES
HC9	male	47	NO	YES
HC10	male	35	NO	YES
HC11	male	48	NO	YES
HC12	female	39	NO	YES
HC13	female	40	NO	YES
HC14	female	31	NO	YES
HC15	female	21	NO	YES
HC16	female	36	NO	YES
